# The AP2 transcription factor *NtERF172* confers drought resistance by modifying *NtCAT*

**DOI:** 10.1111/pbi.13419

**Published:** 2020-06-15

**Authors:** Qiang Zhao, Ri‐Sheng Hu, Dan Liu, Xin Liu, Jie Wang, Xiao‐Hua Xiang, Yang‐Yang Li

**Affiliations:** ^1^ College of Horticulture Qingdao Agricultural University Qingdao China; ^2^ Hunan Tobacco Research Institute Changsha Hunan China; ^3^ Tobacco Research Institute Chinese Academy of Agricultural Sciences Qingdao Shandong Province China; ^4^ Haikou Cigar Research Institution Haikou Hainan Province China

**Keywords:** ERF transcription factor, *NtERF172*, drought stress, catalase activity, H_2_O_2_ levels, transcriptional regulation

## Abstract

Drought stress often limits plant growth and global crop yields. Catalase (CAT)‐mediated hydrogen peroxide (H_2_O_2_) scavenging plays an important role in the adaptation of plant stress responses, but the transcriptional regulation of the *CAT* gene in response to drought stress is not well understood. Here, we isolated an APETALA2/ETHYLENE‐RESPONSIVE FACTOR (AP2/ERF) domain‐containing transcription factor (TF), *NtERF172*, which was strongly induced by drought, abscisic acid (ABA) and H_2_O_2_, from tobacco (*Nicotiana tabacum*) by yeast one‐hybrid screening. *NtERF172* localized to the nucleus and acted as a transcriptional activator. Chromatin immunoprecipitation, yeast one‐hybrid assays, electrophoretic mobility shift assays and transient expression analysis assays showed that NtERF172 directly bound to the promoter region of the *NtCAT* gene and positively regulated its expression. Transgenic plants overexpressing *NtERF172* displayed enhanced tolerance to drought stress, whereas suppression of *NtERF172* decreased drought tolerance. Under drought stress conditions, the *NtERF172*‐overexpressed lines showed higher catalase activity and lower accumulation of H_2_O_2_ compared with wild‐type (WT) plants, while the *NtERF172*‐silenced plants showed the inverse correlation. Exogenous application of amino‐1,2,4‐triazole (3‐AT), an irreversible CAT inhibitor, to the *NtERF172‐*overexpression lines showed decreased catalase activity and drought tolerance, and increased levels of cellular H_2_O_2_. Knockdown *of NtCAT* in the *NtERF172‐*overexpression lines displayed a more drought stress‐sensitive phenotype than *NtERF172‐*overexpression lines. We propose that *NtERF172* acts as a positive factor in drought stress tolerance, at least in part through the regulation of CAT‐mediated H_2_O_2_ homeostasis.

## Introduction

Drought is a major abiotic stressor with several adverse effects on plants, including stomatal closure, metabolic and osmotic damage, repression of cell growth and photosynthesis (Flexas and Medrano, 2002; Tardieu *et al.*, 2014). It limits the growth, productivity and quality of crops (Farooq *et al.*, 2009; Thirumalaikumar *et al.*, 2017). Plants have evolved a variety of complex mechanisms to respond and adapt to drought stress through biochemical and physiological processes, such as reducing water loss and increasing tolerance (Basu *et al.*, 2016; Osakabe *et al.*, 2014; Rasheed *et al.*, 2016).

Previous studies in *Arabidopsis*, rice and other plants have shown that drought stress increases the production of reactive oxygen species (ROS), including the singlet oxygen (^1^O_2_), superoxide anions ( $O_{2}^{\cdot -}$), hydrogen peroxide (H_2_O_2_) and hydroxyl radicals (OH). ROS overaccumulation in cells results in oxidative damage to DNA, RNA, proteins and membranes (Choudhury *et al.*, 2016; Miller *et al.*, 2010; Mittler, 2017). Therefore, plants have evolved both enzymatic and nonenzymatic defence systems for maintaining ROS at non‐damaging levels. In enzymatic systems, enzymes such as superoxide dismutase (SOD), peroxidase (POD), catalase, ascorbate peroxidase (APX) and glutathione peroxidase (GPX) are the major ROS scavengers for maintaining overall plant homeostasis (Mittler, *et al.*, 2004). In contrast to other peroxidases, catalase (EC 1.11.1.6), which requires a cofactor to catalyse the dismutation of H_2_O_2_ into water (H_2_O) and O_2_, plays important role in plant growth and response to environment factors (Mhamdi *et al.*, 2010). Catalases are highly expressed and have a very rapid turnover rate because the affinity (*K*_m_, 40–600 mm) is much lower than that of APX and peroxiredoxin (PRX) (*K*_m_, 100 µm) for H_2_O_2_ (Chelikani *et al.*, 2004; König *et al.*, 2002).

In animals, *CAT* is encoded by a single gene. However, in most other organisms, catalase enzymes are encoded in multiple genes. In plants, *CATs* are encoded by a small gene family and have been reported in *Arabidopsis*, tobacco, maize, rice and tomato (Du *et al.*, 2008; Mhamdi *et al.*, 2010; Wang *et al.*, 2019). Studies have demonstrated that the various forms of *CAT* genes can exhibit different expression patterns, functions and cellular localization in some plants. In *Arabidopsis*, there are three gene members, *CAT1* (At1g20630), *CAT2* (At4g35090) and *CAT3* (At1g20620), which share high sequence similarity and are involved in catalysing H_2_O_2_ (Du *et al.*, 2008; Frugoli *et al.*, 1996). *CAT1* is mainly found in rosette leaves and siliques, *CAT2* is primarily expressed in young leaves, siliques and flowers, and *CAT3* is expressed in all tissues, with a stronger expression in roots and young leaves (Du *et al.*, 2008; Mhamdi *et al.*, 2010; Zimmermann *et al.*, 2006). Catalase activity or expression is induced by various stresses (including salinity, drought, cold and H_2_O_2_) (Du *et al.*, 2008; Leung, 2018). In potatoes, the *CAT* gene was induced in roots by nematodes and bacteria (Niebel *et al.*, 1995). *NtCAT2* mRNA expression in tobacco was induced by treatment with tobacco mosaic virus or fungal elicitor (Dorey *et al.*, 1998). In *Arabidopsis*, no obvious phenotype was detected in *cat1* and *cat3* knockout mutants, while *cat2* had a dwarf phenotype (Mhamdi *et al.*, 2010). However, ectopic expression of the maize *CAT2* gene in tobacco enhanced the resistance to pathogen infection (Polidoros *et al.*, 2001). In rice, overexpressed *CatA* and *CatC* improved drought stress tolerance in transgenic rice (Joo *et al.*, 2014). The *cat3* mutant showed sensitivity to drought stress and increased *CAT3* expression leading to enhanced tolerance to drought stress in *Arabidopsis* (Zou *et al.*, 2015). Knockout of *CAT2* reduced catalase activity by 80% and *cat2* mutant plants were hypersensitive to H_2_O_2_, NaCl and cold (Bueso *et al.*, 2007; Juul *et al.*, 2010).

The regulation mechanisms of CAT have been investigated at the transcriptional and translational levels under different stresses. Several proteins have been reported to interact with CAT protein or regulate *CAT* expression. For instance, receptor‐like cytoplasmic kinase 1 (STRK1) interacted with and phosphorylated CatC, improving the salt tolerance in rice (Zhou *et al.*, 2018). CALCIUM‐DEPENDENT PROTEIN KINASE8 (CPK8) could phosphorylate CAT3 and functioned in ABA‐mediated stomatal regulation in response to drought stress (Zou *et al.*, 2015). In addition, ABA‐INSENSITIVE 5 (ABI5) directly bound to the *CAT1* promoter and regulated seed germination by affecting ROS homeostasis in *Arabidopsis* (Bi *et al.*, 2017). G‐BOX‐BINDING FACTOR 1 (GBF1) directly regulated *CAT2* transcription to promote pathogen defence in *Arabidopsis* (Giri *et al.*, 2017). However, it is not clear whether or how the TF proteins are involved in enhancing drought tolerance by binding the promoter of *CATs* and regulating CAT‐mediated ROS scavenging. In this study, we report on an AP2 TF, NtERF172, which binds to the *NtCAT* promoter and play an important role in maintaining H_2_O_2_ homeostasis in response to drought stress.

## Results

### Involvement of the catalase gene *NtCAT* in drought tolerance

**Figure 1:**
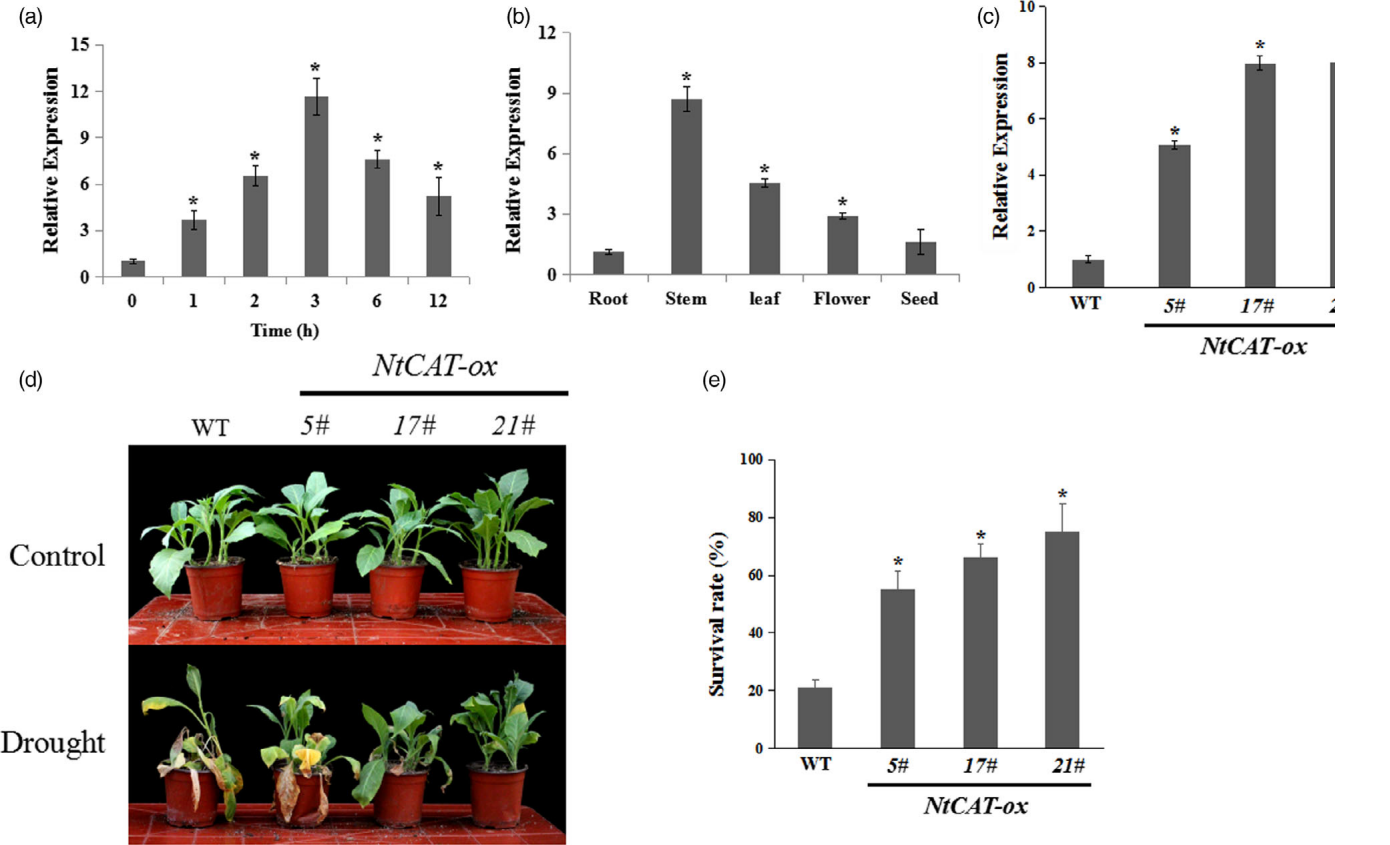
*NtCAT* acts as a positive regulator in response to drought stress. (a) Expression of *NtCAT* in response to drought. (b) Detection of *NtCAT* transcript in different tissues of tobacco plants. Asterisks indicate a significant difference relative to root (*P < 0.01). (c) qRT‐PCR analysis of *NtCAT* expression in *NtCAT‐*ox lines. (d) Drought stress sensitivity of the wild‐type (WT) and *NtCAT‐*ox lines (5#, 17# and 21#) in soil after water was withheld for 15 day. The experiments were repeated three times with similar results. (e) Survival rates of the WT and the transgenic lines after rewatering for 7 day. The experiments were repeated three times. Each data point represents mean ± SD. Asterisks indicate a significant difference relative to WT (*P < 0.01).

To confirm whether *NtCAT* functioned in response to drought stress (see Figure S1a in Supporting Information), we examined the expression of NtCAT mRNA in response to drought treatment using quantitative real‐time reverse transcription PCR (qRT‐PCR). The *NtCAT* transcript level was slightly induced within 1 h and peaked at 3 day, suggesting that the expression of *NtCAT* was induced by drought (Figure 1a). The tissue‐expression patterns of *NtCAT* were analysed using qRT‐PCR. The results showed that *NtCAT* was more abundant in the stem, leaf and flower than in the root and seed (Figure 1b). Then, the wild‐type (WT) and *NtCAT* overexpression (*NtCAT‐*ox, 5#, 17# and 21#) lines were tested for their drought tolerance (Figure 1c). After a 15‐d period of drought stress treatment, the *NtCAT‐*ox lines showed enhanced tolerance to drought stress compared with WT plants (Figure 1d). Consistent with these results, *NtCAT‐*ox plants showed a higher survival rate of 55%–70% compared to that of the WT plants (21%) (Figure 1e).

### Identification of the key region(s) and TF(s) binding sites in the *NtCAT* promoter

In order to identify the *cis*‐regulatory sequences required for drought response, we performed a series of 350 bp‐deleted promoter constructs fused to the β‐glucuronidase (GUS) reporter gene in transgenic tobacco (namely, Δ1, Δ2, Δ3, Δ4 and Δ5, Figure S1b, c). As shown in Figure S1d, the GUS activities were enhanced under drought stress treatment in the full‐length (FL) (−2700 to 1), Δ1 (−2350 to 1) and Δ2 (−2000 to 1) plants. However, the deletion from position Δ3 (−1650 to 1), Δ4 (−1300 to 1) and Δ5 (−2700 to −2000 and −1650 to 1) lost the response to drought stress treatment.

To identify the TF(s) that regulated the expression of *NtCAT* and the activity of catalase under drought stress, a yeast one‐hybrid (Y1H) system was carried out against a cDNA library generated from tobacco using the P sequence as a bait. The clones were screened by SD/‐Leu/‐Trp/‐His with 150 mm AbA. Among several positive clones, an AP2 domain protein, NtERF, was eventually identified as a binding protein. The open reading frame (ORF) of NtERF was 1035 nucleotides and encoded a protein of 344 amino acid. Sequence analysis showed that NtERF contained the highly conserved AP2/ERF domain putatively involved in DNA binding (Figure S2a). Phylogenetic tree analysis of NtERF in relation to AP2 members from tobacco and other plant species showed NtERF with *Arabidopsis* ERF17, but sharing an approximately 100% sequence identity with NtERF172 (the gene named by Rushton *et al.*, 2008), so the gene encoding this TF was named *NtERF172* (Figure S2b).

### *NtERF172* expression is significantly induced by drought stress

**Figure 2:**
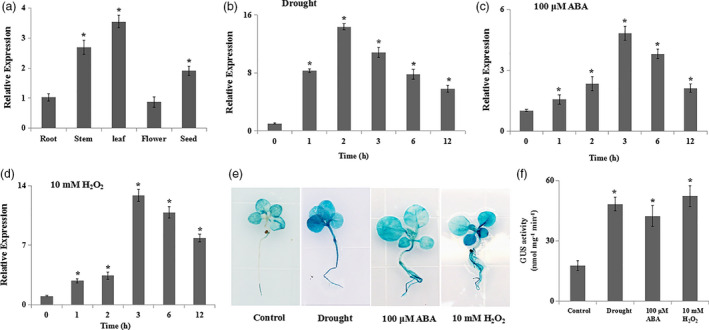
The expression profiles of *NtERF172* in response to drought stress, abscisic acid (ABA) and hydrogen peroxide (H_2_O_2_). (a) Expression of *NtERF172* in the different tissues. Values are means ± SD of three biological replicates. Asterisks indicate a significant difference relative to root (*P < 0.01). (b‐d). Expression analyses of *NtERF172* by quantitative real‐time PCR (qRT‐PCR). Tobacco plants were treated with drought, 100 μm ABA and 10 mm H_2_O_2_ for the indicated times. Data represent means ± SD from three biological replicates. (d, e) Analyses of *NtERF172* expression in response to drought, ABA and H_2_O_2_ treatment for 4h using β‐glucuronidase (GUS) histochemical staining (d) and GUS activity measurement (e). Three independent replicates were performed, and data represent means ± SD of at least three biological replicates. Asterisks indicate a significant difference relative to control (*P < 0.01).

The tissue‐expression patterns of *NtERF172* were first detected by qRT‐PCR. *NtERF172* was expressed in all tissues including root, stem, leaf, flower and seed, but with higher expression in stem, leaf and seed (Figure 2a). To assess the expression of *NtERF172* in response to drought stress, we performed qRT‐PCR using RNA isolated from drought‐treated tobacco. *NtERF172* was significantly induced by drought stress (Figure 2b). Drought stress is known to induce the accumulation of various signal molecules, such as ABA and H_2_O_2_. Therefore, we examined the transcription of *NtERF172* following treatment with 100 μm ABA and 10 mm H_2_O_2_. qRT‐PCR analysis revealed that ABA and H_2_O_2_ treatment significantly up‐regulated *NtERF172* expression, with the highest increases (sixfold and 13.8‐fold, respectively) at 3 h after treatment (Figure 2c, d).

To further analyse the expression of *NtERF172*, a 2.1‐kb fragment upstream from the initiation codon was cloned into pCXGUS‐P vector and transformed into tobacco plants (Figure S2c). GUS staining and GUS activity measurements consistently showed increased GUS activity in response to drought, ABA and H_2_O_2_ treatments in both the leaves and roots of plants expressing *P_NtERF172_::GUS* (Figure 2e, f). These results suggest that the *NtERF172* transcript was strongly induced by drought, ABA and H_2_O_2_.

### Subcellular localization and transcriptional activity analysis of NtERF172

To verify the subcellular localization of the NtERF172 protein, we generated a construct (*35S::NtERF172‐GFP*) by fusing the GFP reporter protein in the C‐terminal under the cauliflower mosaic virus 35S promoter (CaMV 35S). The constructs were expressed in *N. benthamiana* epidermal cells by *Agrobacterium tumefaciens*‐mediated transformation. The vector *35S::GFP* was used as a control. A strong green fluorescence signal was detected only in the nucleus, which was confirmed by staining with DAPI, indicating that NtERF172 was a nuclear protein (Figure S3a).

The transcriptional activity of TFs has been reported to be an important feature. To investigate whether this was also true for NtERF172, a yeast two‐hybrid assay was used. We fused the full‐length NtERF172 with a GAL4 DNA‐binding domain in the pGBKT7 vector to form recombinant vector pGBKT7‐NtERF172, using pGBKT7 as a negative control. The constructs were transformed into the yeast strain Y2H, which were then screened on the selection medium (SD/‐Trp/‐His/‐Ade). The yeast cells grew well on SD/‐Trp medium, whereas only the cells transformed with the recombinant vector pGBKT7‐NtERF172 survived on the selection medium alone (Figure S3b), suggesting that NtERF172 possesses transcriptional activity.

### NtERF172 binds to the promoter of *NtCAT*

**Figure 3:**
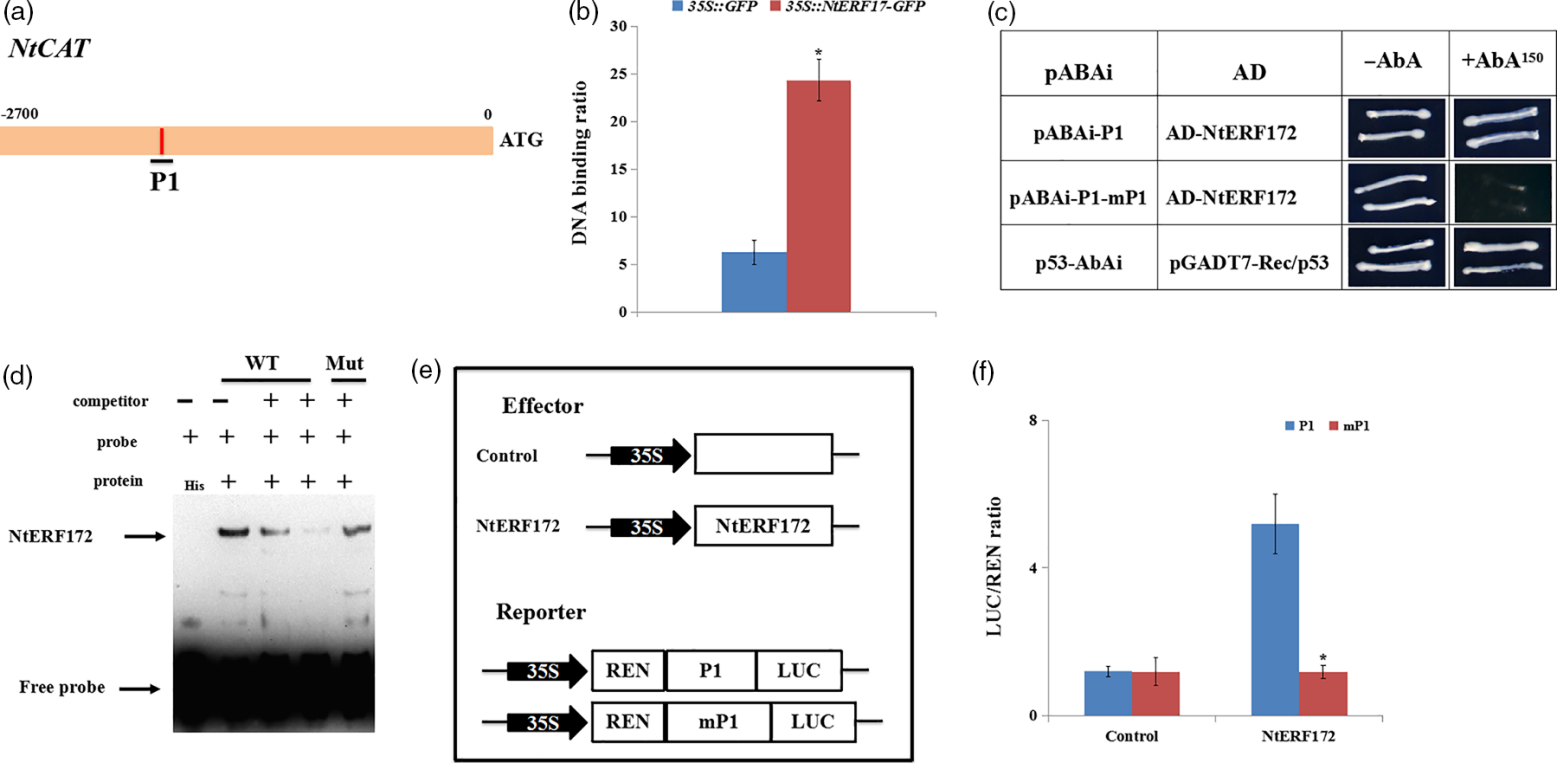
NtERF172 binds to the promoter regions of *NtCAT* gene. (a) Schematic structures of the motif in *NtCAT* promoter (P1 indicates the putative DRE motif). (b) Chromatin immunoprecipitation (ChIP)‐PCR shows that NtERF172 binds to the DRE motif of the *NtCAT* promoter *in vivo*. (c) Y1H Gold yeast cells of bait‐prey co‐transformation on SD/‐Leu medium with or without 150 ng/mL AbA. P1 (GCCGACGT) indicates the fragment with the normal motif sequence. mP1 (GCTTTTGT) indicates the mutant motif sequence. (d) His‐NtERF172 bound to the promoter regions of *NtCAT* as determined by EMSA analysis. Arrows indicate protein–DNA complexes (upper arrows) or free probe (lower arrows). WT, unlabelled probes. Mut, DRE‐mutated probes. His was used as the control. ‘+’ indicates presence, and ‘–’ indicates absence. (e) Schematic of the reporters and effectors used in the transient expression assays. (f) Transient expression assays of the promoter activities co‐transformed with effector and reporter constructs using P1 or mP1. Error bars indicate ± SD of at least three biological replicates. Asterisks indicate a significant difference relative to WT (*P < 0.01).

One DRE motif (P1) was found in the promoter of *NtCAT* (from −2000 to −1650, Figure 3a). To confirm that NtERF172 could directly bind to the DRE motif sequence in the promoter of *NtCAT*, we performed chromatin immunoprecipitation (ChIP)‐PCR, Y1H assays and an electrophoretic mobility shift assay (EMSA). In the ChIP‐PCR assay, the *35S::GFP* and *35::NtERF172‐GFP* fusion proteins were expressed in tobacco plants and immunoprecipitated using an anti‐GFP antibody. The *NtCAT* bands were amplified when *35::NtERF172‐GFP* was precipitated, but the corresponding DNA fragments in the *35S::GFP* control were not (Figure 3b). This demonstrated that NtERF172 could bind to the *NtCAT* promoter regions around the P1 elements *in vivo*. In the Y1H assay, all the yeast cells grew normally on the SD/‐Leu medium without 150 ng/mL AbA. However, when 150 ng/mL AbA was added, growth continued in the positive control and bait‐prey, whereas the mP1 and AD‐NtERF172 were completely inhibited (Figure 3c). This indicated that NtERF172 interacted with the promoter fragment in yeast. In the EMSA, a band was detected for His‐NtERF172, but not for the His protein. The band was reduced when increasing amounts of the unlabelled P1 competitor probe with the same sequence were added. This competition was not observed when the mutated version was used (Figure 3d).

To examine whether NtERF172 directly activated the *NtCAT* promoter, we performed transient expression assays using a double reporter plasmid containing firefly luciferase (LUC) driven by promoter fragments containing P1 or mP1, and *Renilla* luciferase (REN) driven by the 35S promoter, together with an effector plasmid expressing NtERF172. The LUC/REN ratio was significantly lower in the controls than in the presence of NtERF172 (Figure 3e), indicating that NtERF172 directly activated the promoter fragment. However, when the motifs were mutated, the LUC/REN ratios of the samples were similar to those of the controls, indicating that no activation was observed (Figure 3f).

### Overexpression of *NtERF172* increases drought resistance

To further understand the function of *NtERF172*, we transformed the *35S::NtERF172* construct into tobacco (*Nicotiana tabacum* L. ‘NC89’) by *A. tumefaciens‐*mediated leaf disc transformation. Three T3 representative *NtERF172*‐ox lines (designated L1, L2 and L3) were selected using qRT‐PCR for further analysis (Figure S4a). The effects of PEG6000 treatment on seed germination were examined. Under normal conditions, transgenic lines and WT plants exhibited similar germination rates (Figure S4b). When the medium was supplemented with 10% PEG6000, the seed germination ratios of WT and the three transgenic lines significantly decreased. However, the three transgenic lines exhibited higher seed germination ratios than WT. Furthermore, over 85% of the WT seeds failed to germinate, whereas approximately 50%–65% of the seeds of the three transgenic lines germinated under 15% PEG6000 treatment (Figure S4b).

**Figure 4:**
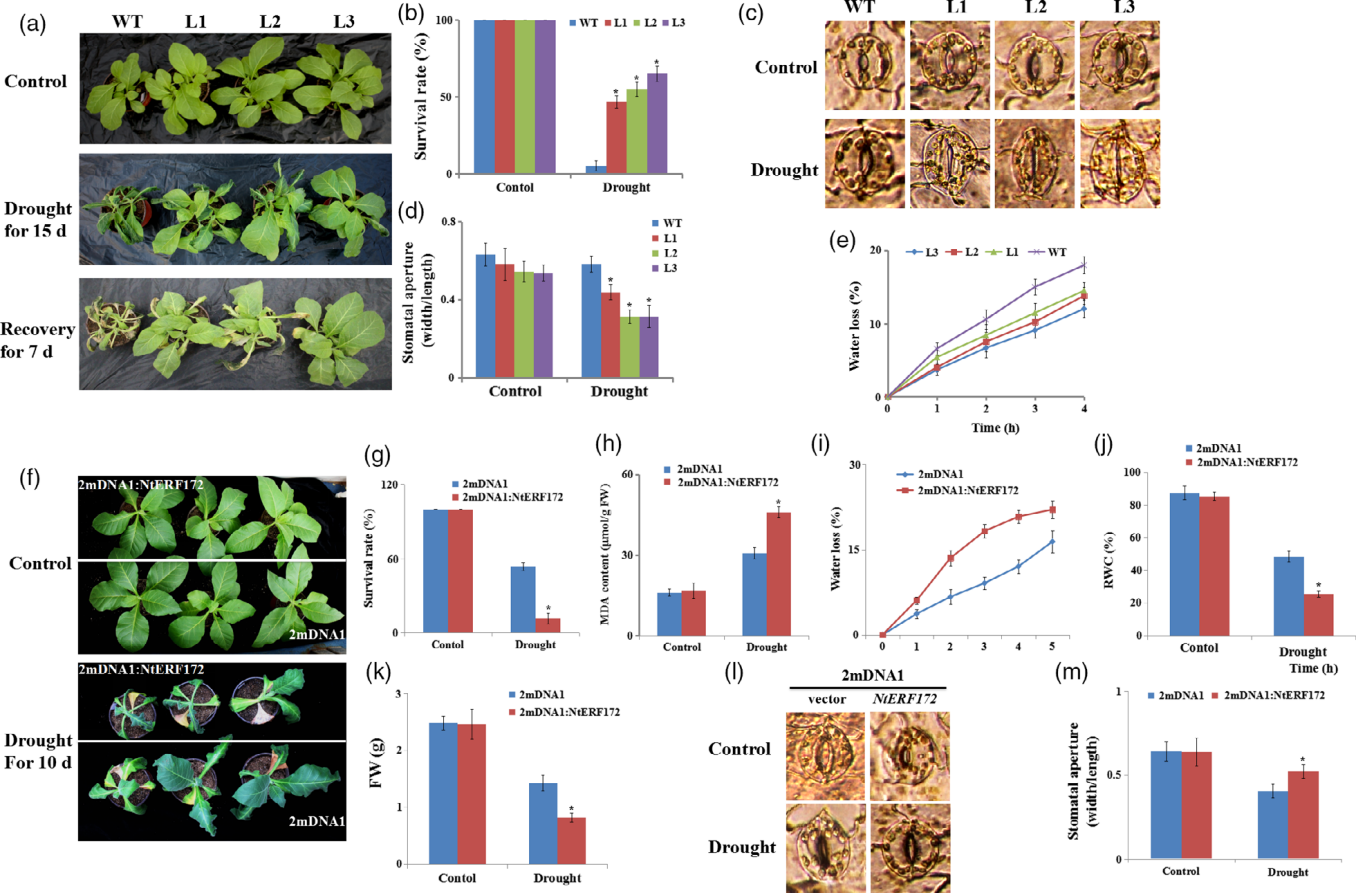
Analysis of the drought tolerance in *35S::NtERF172* and *NtERF172*‐silenced plants. (a) Drought resistance in *35S::NtERF172* transgenic plants (L1, L2 and L3). Wild‐type (WT) and *35S::NtERF172* plants were grown in soil with sufficient water for 2 weeks before water was withheld for 15 days, followed by recovery for 7 days. (b) Survival rates of WT and *35S::NtERF172* transgenic plants were investigated during recovery watering for 7 days. Average survival rates and standard errors were calculated from at least three biological replicates. (c, d) Stomatal closure in WT and *35S::NtERF172* transgenic plants under drought conditions. Values are mean ratios of width to length. Error bars represent the SE of at least three biological replicates. (e) Water loss from detached leaves of WT and *35S::NtERF172* transgenic plants. Water loss is expressed as the percentage of initial fresh weight (FW). Values are means of six leaves each from at least three biological replicates. Asterisks indicate a significant difference relative to WT (*P < 0.01). (f) Phenotypic comparison of tobacco plants subjected to drought stress. 2mDNA1 and *NtERF172*‐silenced plants (2mDNA1:NtERF172) were grown in soil with sufficient water before water was withheld for 10 days. (g‐k) Survival rates (g), malondialdehyde (MDA) content (h), water loss (i), RWC (j) and FW (k) in tobacco plants measured after drought stress treatment. RWC, relative water content. FW, fresh weight. Each bar represents the mean of at least three biological replicates. (l, m) Stomatal closure of 2mDNA1 and *NtERF172*‐silenced plants under control or drought stress conditions. Data represent means ± SD (*n* ≥ 30). Asterisks indicate a significant difference relative to WT (*P < 0.01).

The transgenic tobacco lines were morphologically indistinguishable from WT plants under normal growing conditions. To investigate the potential role of *NtERF172* in drought resistance, water was withheld from 30‐day‐old WT and *35S::NtERF172* transgenic lines for 15 days. WT plants exhibited wilting symptoms, whereas the transgenic lines showed little damage. After 7 days of recovery watering, WT plants wilted and their leaves turned dry, whereas most *35S::NtERF172* transgenic plants remained turgid and their leaves remained green (Figure 4a). Approximately half of the transgenic plants survived (40.5%–62.3%), whereas the survival rate of the WT plants was only 18.3% (Figure 4b). These results suggested that the *35S::NtERF172* transgenic plants may have reduced their daily transpiration rate.

We next compared the stomatal apertures of leaves of *35S::NtERF172* transgenic lines and WT plants. Under normal conditions, there were no differences in the stomatal aperture index between WT and *35S::NtERF172* transgenic lines. However, following drought treatment, the stomatal apertures of *35S::NtERF172* transgenic lines were significantly smaller than those of WT plants (Figure 4c, d). Consistent with these results, the rate of water loss from the detached leaves of WT plants was much faster than that of *35S::NtERF172* transgenic lines (Figure 4e). This suggests that the differences in drought tolerance between WT and *35S::NtERF172*‐overexpressing lines are at least partly attributable to the inability of WT plants to efficiently close their stomata and reduce transpiration.

We then measured the relative water content (RWC) of the leaves. Under normal conditions, the RWC was similar between the transgenic lines and WT. Following drought treatment, the RWC in transgenic lines decreased to about 58%–67%, but it decreased to approximately 38% in WT (Figure S5a), demonstrating that transgenic lines had higher RWC compared with WT. Additionally, we measured levels of ion leakage (IL) and malondialdehyde (MDA, as a lipid peroxidation marker), which are important indicators of membrane damage (Moore and Roberts, 1998). Under drought stress, transgenic lines had significantly lower MDA and IL accumulation compared to WT (Figure S5b, c), suggesting that the transgenic plants suffered less membrane damage than WT plants during drought stress. The fresh weight (FW) did not differ significantly between WT and transgenic plants under control conditions, but transgenic lines had significantly higher FW than WT under drought stress conditions (Figure S5d). These physiological parameters demonstrate that the transgenic lines were more resistant to drought stress.

### *NtERF172*‐silenced plants are more sensitive to drought stress

To further elucidate the role of *NtERF172* in drought tolerance, we created *NtERF172*‐silenced plants using a virus‐induced gene silencing (VIGS) system in tobacco. In silencing construct‐infected plants (2mDNA1:NtERF172), *NtERF172* transcripts were significantly reduced compared with controls infected with empty vector (2mDNA1) (Figure S6a). After a 10‐day drought period, the *NtERF172*‐silenced plants suffered more severe injury than the 2mDNA1 plants. Of the *NtERF172*‐silenced plants, only 11.6% survived, whereas the survival rate of the empty vector plants was 53.2% (Figure 4f, g). The 2mDNA1 plants exhibited a significantly lower MDA content relative to the *NtERF172*‐silenced plants (Figure 4h). The rate of water loss from detached leaves under drought conditions was lower in 2mDNA1 plants than in *NtERF172*‐silenced plants (Figure 4i). Additionally, the RWC and FW in *NtERF172*‐silenced plants were significantly lower compared to 2mDNA1 plants (Figure 4j, k). The stomatal apertures of *NtERF172*‐silenced plants were more open than those of 2mDNA1 plants after drought treatment (Figure 4l, m). These results suggest that silencing *NtERF172* reduced drought tolerance in tobacco seedlings.

### *NtERF172* regulates H_2_O_2_ accumulation and catalase enzyme activities

**Figure 5:**
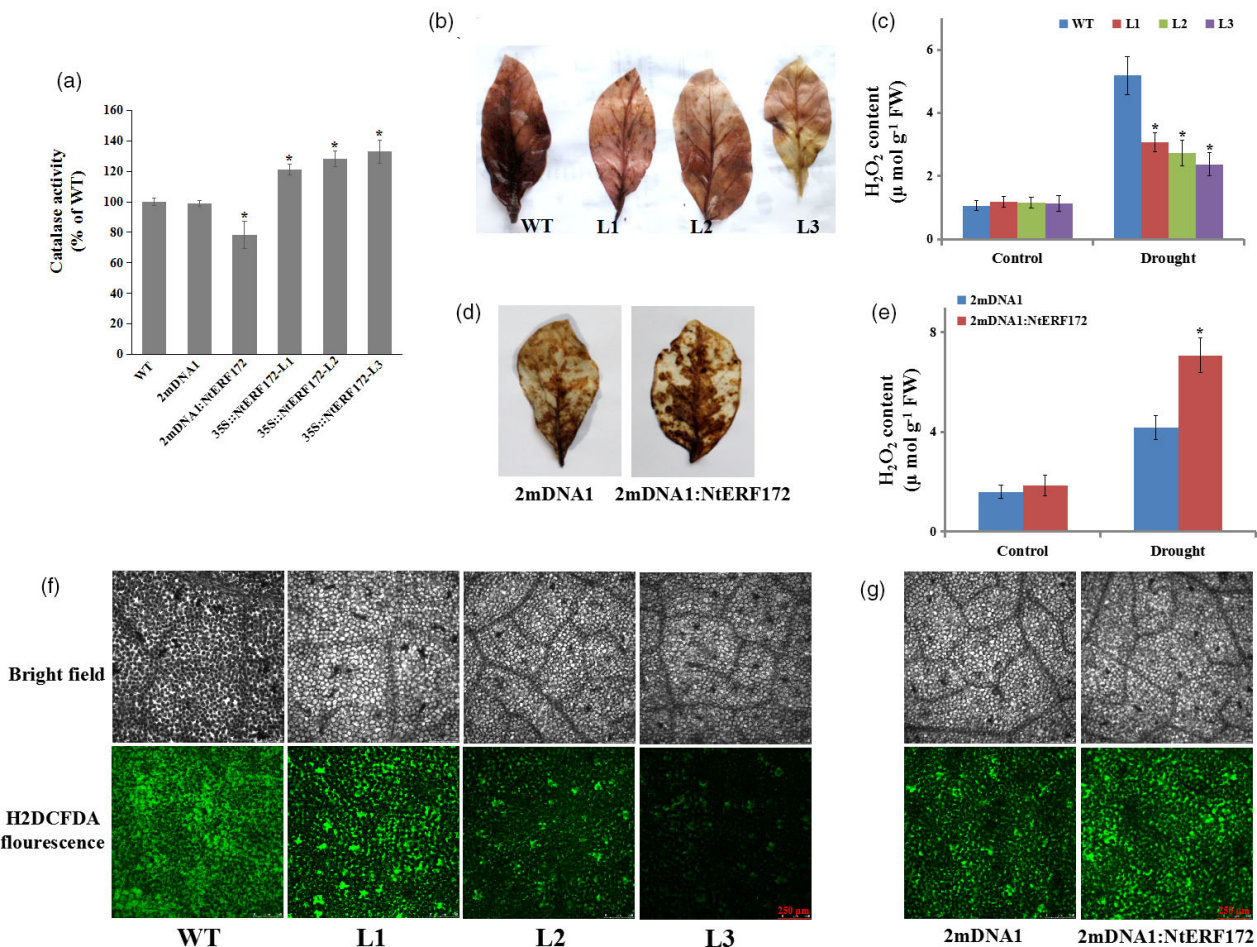
Comparison of catalase activity and reactive oxygen species (ROS) accumulation in overexpression and silenced plants. (a) Catalase activity in WT, 2mDNA1, 2mDNA1:NtERF172 and *35S::NtERF172*‐ox (L1, L2 and L3) plants. Each data point represents mean ± SD (*n* = 3). (b, c) Effects of drought stress on H_2_O_2_ accumulation in both WT and *35S::NtERF172* transgenic lines under drought conditions. Data represent the means of at least three biological replicates (± SD). (d, e) Histochemical staining and quantitative measurement of H_2_O_2_ in 2mDNA1 and *NtERF172*‐silenced plants after drought treatment. Data represent the means of at least three biological replicates ± SD. Asterisks indicate a significant difference relative to WT (*P < 0.01). (f, g) ROS accumulation of leaves detected using the ROS‐sensitive fluorescent probe H2DCFDA in WT, 2mDNA1, 2mDNA1:NtERF172 and *35S::NtERF172*‐ox (L1, L2 and L3) plants under drought conditions; bar = 250 µm.

Since NtERF172 could bind to the *NtCAT* promoter and activate its transcription, it was possible that the increased drought tolerance of the transgenic line might be associated with *NtCAT*‐mediated ROS‐scavenging ability. As shown in Figure 5a, the activities of the catalase enzyme, a product of *NtCAT*, were higher in the *35S::NtERF172* (the representative line L3 was selected for catalase enzyme activity) transgenic line than in the WT, but the *NtERF172*‐silenced plants had a decrease in catalase activity of 22.3% compared with WT plants.

We, therefore, examined the accumulation of H_2_O_2_ and ROS. Drought stress increased H_2_O_2_ levels in WT and transgenic, but with an accelerated accumulation in WT, as indicated by 3,3ʹ‐diaminobenzidine (DAB) staining and measurement (Figure 5b, c). H_2_O_2_ accumulation was higher in *NtERF172*‐silenced plants than in 2mDNA1 plants under drought treatment (Figure 5d, e). The ROS production in leaves of *NtERF172‐*ox, *NtERF172*‐silenced, 2mDNA1 and WT plants was further analysed using the dye 2′,7′‐dichlorofluorescein diacetate (H2DCF‐DA) (Bao *et al.*, 2015). After treatment with drought stress, the pixel intensity of fluorescence emission was lower in the leaves of *NtERF172‐*ox plants and higher in *NtERF172*‐silenced plants than those of WT plants and 2mDNA1, respectively (Figure 5f, g). All these data indicate that *NtERF172* can mediate the ROS accumulation and catalase activity under drought stress conditions.

### Decreased catalase activity suppresses the drought tolerance phenotype of *NtERF172‐*ox plants

**Figure 6:**
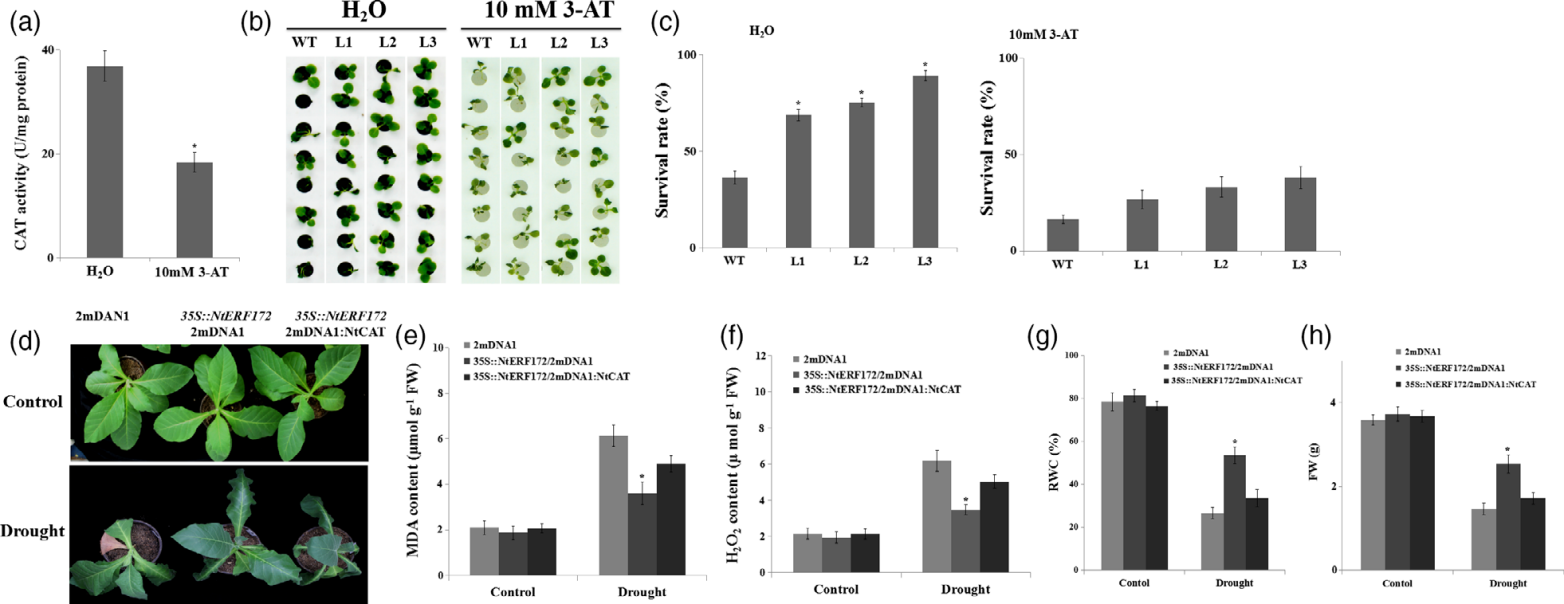
Silenced *NtCAT* represses the drought tolerance of *NtERF172‐*ox plants. (a) Catalase activity in plants treated with water or 10 mm 3‐AT for 5 h. (b) Phenotypes of PEG6000‐treated transgenic and WT plants pretreated with water or 10 mm 3‐AT. The treatment was repeated five times with ten replicates for different lines at each repetition. (c) Survival rates of transgenic and WT plants were analysed following PEG6000 treatment. The data represent the means ± SD of at least three biological replicates. Asterisks indicate a significant difference relative to WT (*P < 0.01). (d) The drought phenotypes of 2mDNA1, *35S::NtERF172*/2mDNA1 and *35S::NtERF172*/2mDNA1:NtCAT plants. MDA levels (e), H_2_O_2_ contents (f), RWC (g) and FW (h) of the 2mDNA1, *35S::NtERF172*/2mDNA1 and *35S::NtERF172*/2mDNA1:NtCAT plants after drought treatment. RWC, relative water content. FW, fresh weight.

Based on the above data, we hypothesized that CAT‐mediated ROS scavenging is important for the drought tolerance of the *35S::NtERF172* overexpression plants. To confirm the regulatory pathway, we used 10 mm 3‐amino‐1,2,4‐triazole (3‐AT) as an inhibitor to suppress catalase activity. In the WT, CAT activity was reduced from 36.8 to 18.4 U/mg protein following 3‐AT treatment (Figure 6a). Next, the WT and transgenic seedlings were treated with water or inhibitors and then exposed to 15% PEG6000. When the seedlings were treated with water, the transgenic plants exhibited much better growth than the WT plants, and more than 75.4%–85.7% of the transgenic seedlings survived 15% PEG6000 treatment compared with only 45.2% of the WT seedlings (Figure 6b, c). However, when 3‐AT was used, the survival rate of the transgenic lines was sharply decreased, being only slightly higher than the WT plants (Figure 6b, c).

We then generated *NtERF172*‐ox/2mDNA1:NtCAT plants by transforming 2mDNA1:NtCAT into *NtERF172*‐ox (Figure S6b). We found that knockdown of *NtCAT* partly reduced the tolerance of the drought stress phenotype of *NtERF172*‐ox, including increasing the MDA content and H_2_O_2_ content. Consistent with the visible phenotype, the RWC and FW in *NtERF172*‐ox plants were decreased by knockdown of *NtCAT* (Figure 6d‐h).

### *NtERF172‐*ox plants enhanced oxidative stress tolerance

**Figure 7:**
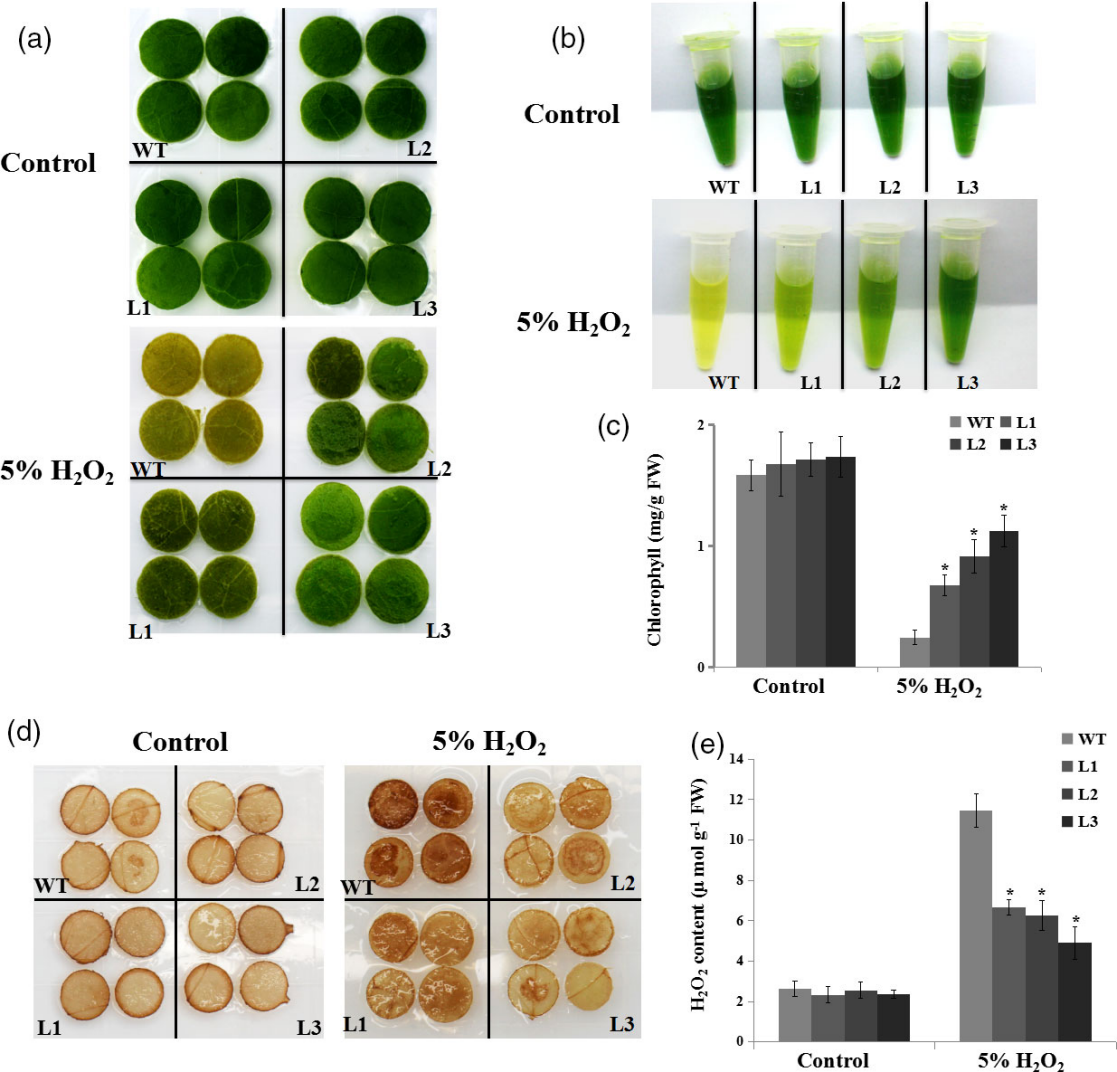
Oxidative stress tolerance assays in WT and *35S::NtERF172* transgenic plants treated with 5% H_2_O_2_. (a) Representative photographs showing leaf pieces of WT and transgenic plants after 5% H_2_O_2_ treatment. (b, c) Chl extraction solutions and Chl content in leaf pieces of WT and *35S::NtERF172* transgenic plants. FW, fresh weight. (d, e) DAB staining and H_2_O_2_ contents of leaves from WT and transgenic plants pretreated with water or 5% H_2_O_2_. Data represent means ± SD calculated from three biological replicates. Three biological experiments produced similar results. Asterisks indicate a significant difference relative to WT (*P < 0.01).

To assess the role of *NtERF172* in regulating oxidative stress, we investigated the tolerance of *NtERF172* caused by H_2_O_2_. Leaves were cut into small pieces and treated for 24 h with distilled water or 5% H_2_O_2_. Under the distilled water treatment conditions, there were no differences between leaf pieces from transgenic lines or WT. However, under 5% H_2_O_2_ treatment, most of the WT pieces turned brown and necrotic, whereas those from transgenic lines maintained their green colour (Figure 7a). The chlorophyll (Chl) content was higher in the transgenic lines than in the WT (Figure 7b, c). Interestingly, the DAB staining and quantitative measurements showed that upon exogenous H_2_O_2_ treatment, the *NtERF172‐*ox line leaves displayed less H_2_O_2_ accumulation than the WT plants (Figure 7d, e), implying that *NtERF172‐*ox leaves had a higher H_2_O_2_‐scavenging capacity. However, the leaf discs from 2mDNA1::NtERF172 transgenic plants had a lower Chl content and higher H_2_O_2_ accumulation compared with 2mDNA1 after 5% H_2_O_2_ treatment (Figure S7).

## Discussion

Drought stress affects physiological metabolic reactions, including oxidative stress and membrane lipids damage through excess ROS accumulation (Fedoroff *et al.*, 2010; Schieber and Chandel, 2014; Zhu, 2016). To protect the cells against damage because of ROS, plants have evolved a broad range of adaptive responses, involving enzymatic and nonenzymatic antioxidants (Miller *et al.*, 2010; Mittler *et al.*, 2004). As an important H_2_O_2_‐scavenging enzyme, the expression or activity of catalase plays a vital role in plant response to stresses (Bueso *et al.*, 2007; Du *et al.*, 2008; Xing *et al.*, 2007; Zimmermann *et al.*, 2006). For example, the transcription of *CAT3* could be induced by drought and overexpression lines were insensitive to drought stress (Zou *et al.*, 2015). In tobacco, biochemical and transcriptomic analyses showed that *CAT* transcript level and catalase enzyme activity were up‐regulated by drought stress treatment (Yang *et al.*, 2017; Yin *et al.*, 2015). The results we obtained are consistent with the view that *NtCAT* expression was induced by drought, and overexpression lines had high survival rates under drought stress conditions (Figure 1), suggesting that *NtCAT* acts as a positive regulator of drought tolerance.

In plants, several TFs have been shown to bind to the promoter regions of *CATs* to activate or suppress their expression and catalase activity involved in seed germination, leaf senescence and disease resistance (Bi *et al.*, 2017; Giri *et al.*, 2017; Smykowski *et al.*, 2010). However, the TFs involved in regulating CAT‐mediated H_2_O_2_ scavenging under drought stress conditions are not well understood. Here, we identified a TF, *NtERF172*, with a potential role as a transcriptional activator in regulating *NtCAT* expression, by screening a cDNA library using the Y1H screening method (Figure 3d). The AP2/ERF family is a large group of plant‐specific TFs that play important roles in plant response to abiotic stresses, including drought, salinity and temperature (Mizoi *et al.*, 2012; Nakano *et al.*, 2006; Sakuma *et al.*, 2002; Xie *et al.*, 2019). Phylogenetic tree analysis indicated that the NtERF172 protein was clustered within the same clade as ERF17 of *Arabidopsis* and belonged to the DREBs subgroup A5 (Figure S2b). The function of *AtERF17* is unclear in terms of stress response, but its homologs from citrus (*CitERF13*) and tomatoes (*SlERF16*) are involved in fruit degreening (Li *et al.*, 2019). From this, we conclude that the NtERF172 of different plant species may exhibit diverse functions. Increasing evidence has shown that ERF transcription levels are regulated by abiotic stress responses (Licausi *et al.*, 2013; Mizoi *et al.*, 2012). For example, the DREB1/CBFs subfamily is rapidly induced in response to cold stress but not to dehydration or high salinity (Jaglo‐Ottosen *et al.*, 1998; Liu *et al.*, 1998). The DREB2 subgroup has eight members which are induced by dehydration, high salinity and heat in an ABA‐independent manner (Mizoi *et al.*, 2012; Xie *et al.*, 2019). The transcript levels of *NtERF172* were also induced by drought treatment (Figure 2), indicating that it may play a role in regulating response to drought stresses.

ERFs are reported to bind to the DRE/CRT or ERE (known as GCC‐box) motifs on stress‐responsive genes to confer resistance to abiotic and biotic stresses (Shinozaki and Yamaguchi‐Shinozaki, 2007). For instance, *AtERF53* overexpression in *Arabidopsis* increases drought tolerance by binding to the GCC‐box and/or the DRE element in the promoters of downstream genes, such as *COR15B* and *P5CS1* (Cheng *et al.*, 2012). *RAP2.4‐*ox *Arabidopsis* enhances drought tolerance by regulating a number of genes containing DRE or a similar *cis*‐element named the C‐repeat (CRT, core sequence TGGCCGAC), including *RD29A*, *COR47* and *COR15A* (Lin *et al.*, 2008). Our work shows that NtERF172, which binds to the DRE motif, is an upstream regulator of *NtCAT*, confirmed by Y1H assay, ChIP‐PCR assay and EMSA (Figure 3). The phenotype analysis indicates that *NtERF172* is a positive regulatory factor in drought stress tolerance in plants (Figure 4). It is well established that antioxidant enzymes, which are regulated by TFs, play a predominant role in eliminating ROS accumulation under abiotic stresses (Choudhury *et al.*, 2016; Mittler, 2017). For example, PtrbHLH binds to the promoter region of a POD gene and positively regulates POD‐mediated ROS removal under cold stress conditions (Huang *et al.*, 2013). AtbHLH112 binds to GCG‐ and E‐box motifs in the promoter regions of SOD or POD genes and mediates ROS scavenging and proline biosynthesis under stress (Liu *et al.*, 2015). ERF74 binds specifically to the GCC‐box element in the promoter of *RbohD* and activates RbohD‐mediated ROS bursts in the early stages of different stresses (Yao *et al.*, 2017). Consistent with these results, *NtERF172* regulated catalase activity and functions in conferring drought tolerance through CAT‐mediated ROS scavenging in the transgenic plants (Figures 5 and 6).

Plants have evolved a variety of mechanisms to improve their drought tolerance (Miller *et al.*, 2010). ERF TFs are involved in several processes in response to drought stress, including stomatal development, root hair formation, root meristem size and hormone metabolism (Shinozaki *et al.*, 2003 Shinozaki and Yamaguchi‐Shinozaki, 2007). Stomatal apertures are a key determinant of transpirational water loss, and stomatal closure reduces water loss. This property is critical for maintaining high water potential under drought stress conditions in plants (Nilson and Assmann, 2007). TF‐mediated stomatal closure is an important process in drought stress responses (Castilhos *et al.*, 2014). *bHLH122* enhances drought tolerance by decreasing stomatal apertures in *Arabidopsis* (Liu *et al.*, 2014). The *Arabidopsis* GTL1 TF regulates water use efficiency by modulating stomatal density under drought stress conditions (Yoo *et al.*, 2010). In this study, *NtERF172* transgenic lines regulated stomatal aperture and transpirational water loss (Figure 4), indicating that *NtERF172* functions as a positive regulator of drought stress responses by regulating stomatal aperture and transpiration.

Based on the results of this study, we propose a model of NtERF172 function in response to drought stress in tobacco plants. Under drought stress conditions, NtERF172 is induced and acts upstream of *NtCAT*, directly regulating its expression by binding to the DRE motif in the promoter region. Activated *NtCAT* then promotes ROS scavenging under drought stress. It is possible that NtERF172 regulates stress via other stress tolerance pathways under drought stress. Additional research is needed to determine whether *NtERF172* directly regulates other stress‐related genes to enhance drought tolerance. We conclude that *NtERF172* exhibits important physiological functions in the drought stress response through the regulation of CAT‐mediated ROS scavenging and other pathways, thus protecting plants against oxidative damage.

## Materials and methods

### Plant materials and treatments

Tobacco cultivars (*N. tabacum* L. ‘NC89’) were used for expression analyses and genetic transformation. The seeds were surface‐sterilized with 3% NaClO for 8 min and then germinated on 1/2 MS medium containing 2.5% sucrose and 1.0% agar at 25°C. The seedlings were transferred to soil and cultivated at 25 ± 1°C with a 16‐h light/8‐h dark photoperiod.

For drought treatment, uniform and healthy 10‐day‐old seedlings were transferred to filter paper and dried at 23°C for 0, 1, 3, 6, 12 and 24 h. For 100 µm ABA, and 10 mm H_2_O_2_ treatments, 10‐day‐old seedlings were removed from the agar medium and submerged in a solution containing 100 µm ABA or 10 mm H_2_O_2_ for the durations indicated. Seedlings were independently harvested and immediately frozen in liquid nitrogen and stored at −80°C until RNA extraction.

### Vector construction and plant transformation

The full‐length sequences of *NtCAT* and *NtERF172* were amplified from tobacco genomic cDNA by PCR and cloned into the binary vector pRI under the control of the CaMV 35S promoter region and a green fluorescent protein (GFP) coding region. For the deletion analysis of the *NtCAT* promoter, the FL (2.7 Kb) and the consecutively truncated regions ∆1 (−2350 to 0 kb), ∆2 (−2000 to 0 kb), ∆3 (−1650 to 0 kb) and ∆4 (−1300 to 0 kb) and ∆5 (−2700 to −2000 kb and −1650 to 0 kb) were amplified by PCR and cloned into pCXGUS‐P to drive the GUS reporter gene. Then, the recombinant plasmids were introduced into the *A. tumefaciens* strain EHA105. The primer sequences are listed in Table S1.

Tobacco transformation was performed by leaf disc transformation using the *A. tumefaciens* method described by Horsch *et al.*(1985). Tobacco leaves were cut into small pieces and immersed in *A. tumefaciens* suspension cultures. Seedlings from three independent T3 homozygous transgenic lines were used in further investigations.

### qRT‐PCR

Total RNAs were isolated from roots, stems, leaves, flowers, seeds and whole tobacco samples using the TRIzol reagent (Invitrogen, Carlsbad, CA) following the manufacturer’s instructions. The first‐strand cDNA was generated with the PrimeScript 1st Strand cDNA Synthesis Kit (TaKaRa, Dalian, China) according to the manufacturer’s protocol. qRT‐PCR was performed using the SYBR Premix Ex Taq (TaKaRa) in a 20‐μL volume and an Applied Biosystems 7500 Real‐Time System (Applied Biosystems, Foster City, CA). The PCR conditions were as follows: 95°C for 30 s, followed by 40 cycles of 95°C for 5 s and 60°C for 34 s. *NtActin* was used as an internal control. The expression analysis at each time point was replicated at least three times. The primer sequences are listed in Table S2.

### Virus‐induced gene silencing assays

The tobacco curly shoot virus‐associated alphasatellite vector (2mDNA1) was used in *N. tabacum* as described by Huang *et al.*(2009). PCR was used to amplify 329‐bp and 220‐bp fragments which were inserted into the plasmid 2mDNA1 to produce 2mDNA1:NtERF172 and 2mDNA1:NtCAT, respectively. Then, the 2mDNA1:NtERF172, 2mDNA1:NtCAT and empty vector (2mDNA1) constructs were transformed into *A*. *tumefaciens* EHA105. The transformed *A. tumefaciens* was individually co‐infiltrated with tomato yellow leaf curl China virus (TYLCCNV) into *N. tabacum* plants as previously described (Huang *et al.*, 2009). *NtERF172* silencing or *NtCAT* silencing was confirmed by qRT‐PCR.

### Germination analysis and stress tolerance assays

The seeds of both WT and transgenic lines were sterilized by 3% NaClO and planted on MS medium or PEG‐infused MS medium (described by Verslues *et al.*, 2006) for tolerance assays and were incubated at 25 ± 1°C with a 16‐h light/8‐h dark photoperiod. The germination rates were measured daily.

For drought tolerance assays, 30‐day‐old soil‐grown WT and transgenic lines were deprived of water for 15 days and then allowed to recover for 7 days. Survival rates were then scored, and physiological indices were measured. Water loss measurements were performed according to Liu *et al.*(2014). Water loss was determined as a percentage of the FW at the beginning of the experiment and weighed at the designated time intervals. IL was measured based on the procedures described by Huang *et al.*(2013). RWC was measured according to Munné‐Bosch *et al.*(1999) and evaluated via the equation RWC (%) = (FW − dry weight)/(turgid weight − dry weight) × 100. The MDA content was detected by the thiobarbituric acid‐based colorimetric method as described by Heath and Packer (1968).

In inhibitor experiments, three‐ or four‐leaf‐stage seedlings were grown in nutrient solutions containing 10 mm 3‐AT for 3 h, using water as a control, before 15% PEG6000 treatment for 24 h. Survival rates were then measured. The activities of endogenous CAT were measured after 10 mm 3‐AT treatment for 6 h.

For the oxidative stress test, leaf pieces from WT and transgenic lines were incubated in 5% H_2_O_2_ for 48 h. After treatment, photographs were taken and total Chl content was measured.

### Stomatal aperture analysis

Stomatal apertures were measured as described previously (Liu *et al.*, 2014). The leaves, which were chosen from uniform and healthy 30‐day‐old WT and transgenic lines, were exposed to drought conditions for 15 or 10 days. The stomata on epidermal strips obtained from the leaves were then measured under a microscope (Olympus ix71, Tokyo, Japan). More than 40 guard cells from each sample were imaged. The width and length of stomatal pores were determined using ImageJ software (National Institutes of Health, Bethesda, MD), and the stomatal apertures were calculated as the ratio of width to length.

### Histochemical staining and catalase activity assays

H_2_O_2_ accumulation in leaves was examined visually using histochemical staining with DAB. The H_2_O_2_ content was quantified according to the method of Zhao *et al.*(2016a). For enzyme assays, catalase activities were measured according to the methods of Zou *et al.*(2015). Each assay was replicated at least three times.

### GUS analysis

For histochemical staining, the transgenic lines and WT were immersed in GUS staining buffer as previously described (Zhao *et al.*, 2016a).

GUS activity analysis was conducted as previously described (Jefferson *et al.*, 1987). The proteins from whole tobacco plants were extracted with extraction buffer and reacted with 4‐MUG (Sigma‐Aldrich, St. Louis, MO) at 37°C. The GUS activity was determined using a VersaFluor spectrofluorometer (excitation 365 nm and emission 450 nm).

### ChIP‐PCR, Y1H and EMSA

ChIP‐PCR assays were performed as described by Zhao *et al.*(2016b). After immunoprecipitation, recovered chromatin fragments were subjected to qRT‐PCR. The primers are shown in Table S1.

Y1H assays were performed according to the manufacturer’s instructions. The full‐length *NtERF172* was cloned into the pGADT7 vector to generate the construct AD‐NtERF172. The promoter fragments of the *NtCAT* gene containing the binding *cis*‐elements were inserted into the pAbAi vector. Different combinations were co‐transformed into the yeast strain Y1H Gold, and the interactions were examined on SD/‐Leu medium with or without 150 ng/mL AbA.

For EMSAs, the *NtERF172* open reading frame was amplified by PCR and cloned into the pET32a vector. The construct was introduced into *E. coli* BL21 (DE3) to induce recombinant His‐NtERF172 protein. The protein was produced and purified with a nickel‐nitrilotriacetic acid (NiNTA) agarose column. EMSAs were carried out using the LightShift Chemiluminescent EMSA Kit (Thermo Fisher Scientific, Waltham, MA) according to the manufacturer’s protocol. Reaction mixtures containing the protein and probes were then incubated for 30 min at room temperature and separated on 6% polyacrylamide gels in 0.5 × TBE buffer. Finally, the signals were detected using the EMSA Kit and ChemiDoc XRS + (Bio‐Rad).

### Transient expression assays

The *NtCAT* promoter fragments containing the binding motifs were cloned into pGreenII 0800‐LUC to act as the reporter gene. Full‐length *NtERF172* was cloned into the pGreenII 62‐SK to serve as the effector.

The constructs and the pSoup helper were transformed into *A*. *tumefaciens* EHA105 and then infiltrated into tobacco (*N. benthamiana*) leaves. Three days later, the infiltrated leaves were collected and the activities of firefly and REN luciferase were measured using dual luciferase assay reagents (Beyotime Institute of Biotechnology, Haimen, China). Eight independent biological samples were used.

### Statistical analysis

Data analyses were performed using SPSS 16.0 software (SPSS Inc., Chicago, IL). Experimental data were analysed with Student’s *t*‐test with significance at *P* < 0.01 (*). Sample variability is given as the standard deviation (SD) of the mean.

## Conflicts of interest

The authors declare no conflict of interest.

## Author contributions

QZ, XHX and YYL conceived and designed the research. QZ performed most of the experiments. QZ, RSH, DL, XL, JW, XHX and YYL performed the research. QZ, XHX and YYL analysed the data and wrote the paper.

## Supporting information


**Figure S1**. Promoter analysis of the *NtCAT* promoter.
**Figure S2**. Phylogenetic and conserved domain analysis of NtERF172.
**Figure S3**. Subcellular localization and transcriptional activity of NtERF172.
**Figure S4**. Analysis of germination of wild‐type (WT) and *NtERF172‐*ox plants exposed to PEG6000 treatment.
**Figure S5**. Analysis of relative water content (RWC), ion leakage (IL), malondialdehyde (MDA), and fresh weight (FW) in wild‐type (WT) and *NtERF172‐*ox plants under normal and drought conditions.
**Figure S6**. Expression analysis of the *NtERF172* and *NtCAT* genes.
**Figure S7**. Oxidative stress tolerance assays in 2mDNA1 and 2mDNA1::NtERF172 transgenic plants treated with 5% H_2_O_2_.
**Table S1**. Primers used for ChIP‐PCR, Y1H assays, transient expression assays and plant transformation.
**Table S2**. Primers used in real‐time qRT‐PCR.

## References

[pbi13419-bib-0001] Bao, H. , Chen, X. , Sulian, L.V. , Jiang, P. , Feng, J. , Fan, P. , *et al* (2015) Virus‐induced gene silencing reveals control of reactive oxygen species accumulation and salt tolerance in tomato by γ‐ aminobutyric acid metabolic pathway. Plant, Cell Environ. 38, 600–613. 25074245 10.1111/pce.12419

[pbi13419-bib-0002] Basu, S. , Ramegowda, V. , Kumar, A. and Pereira, A. (2016) Plant adaptation to drought stress. F1000Research 5, 1554. 10.12688/f1000research.7678.1 PMC4937719 27441087

[pbi13419-bib-0003] Bi, C. , Ma, Y. , Wu, Z. , Yu, Y.T. , Liang, S. , Lu, K. and Wang, X.F. (2017) *Arabidopsis* ABI5 plays a role in regulating ROS homeostasis by activating CATALASE 1 transcription in seed germination. Plant Mol. Boil. 94, 197–213. 10.1007/s11103-017-0603-y PMC5437177 28391398

[pbi13419-bib-0004] Bueso, E. , Alejandro, S. , Carbonell, P. , Perez‐Amador, M.A. , Fayos, J. , Bellés, J.M. , Rodriguez, P.L. *et al* (2007) The lithium tolerance of the *Arabidopsis cat2* mutant reveals a cross‐talk between oxidative stress and ethylene. Plant J. 52, 1052–1065. 17931347 10.1111/j.1365-313X.2007.03305.x

[pbi13419-bib-0005] Castilhos, G. , Lazzarotto, F. , Spagnolo‐Fonini, L. , Bodanese‐Zanettini, M.H. and Margis‐Pinheiro, M. (2014) Possible roles of basic helix‐loop‐helix transcription factors in adaptation to drought. Plant Sci. 223, 1–7. 24767109 10.1016/j.plantsci.2014.02.010

[pbi13419-bib-0006] Chelikani, P. , Fita, I. and Loewen, P.C. (2004) Diversity of structures and properties among catalases. Cell. Mol. Life Sci. 61, 192–208. 14745498 10.1007/s00018-003-3206-5 PMC11138816

[pbi13419-bib-0007] Cheng, M.C. , Hsieh, E.J. , Chen, J.H. , Chen, H.Y. and Lin, T.P. (2012) *Arabidopsis* RGLG2, functioning as a RING E3 ligase, interacts with AtERF53 and negatively regulates the plant drought stress response. Plant Physiol. 158, 363–375. 22095047 10.1104/pp.111.189738 PMC3252077

[pbi13419-bib-0008] Choudhury, F.K. , Rivero, R.M. , Blumwald, E. and Mittler, R. (2016) Reactive oxygen species, abiotic stress and stress combination. Plant J. 90, 856–867. 27801967 10.1111/tpj.13299

[pbi13419-bib-0009] Dorey, S. , Baillieul, F. , Saindrenan, P. , Fritig, B. and Kauffmann, S. (1998) Tobacco class I and II catalases are differentially expressed during elicitor‐induced hypersensitive cell death and localized acquired resistance. Mol. Plant Microbe In. 11, 1102–1109.

[pbi13419-bib-0010] Du, Y.Y. , Wang, P.C. , Chen, J. and Song, C.P. (2008) Comprehensive functional analysis of the catalase gene family in *Arabidopsis thaliana* . J. Integr. Plant Biol. 50, 1318–1326. 19017119 10.1111/j.1744-7909.2008.00741.x

[pbi13419-bib-0011] Farooq, M. , Wahid, A. , Kobayashi, N. , Fujita, D. and Basra, S.M.A. (2009) Plant drought stress: effects, mechanisms and management. Agron. Sustain. Dev. 29, 185–212.

[pbi13419-bib-0012] Fedoroff, N.V. , Battisti, D.S. , Beachy, R.N. , Cooper, P.J. , Fischhoff, D.A. , Hodges, C.N. *et al* (2010) Radically rethinking agriculture for the 21st century. Science 327, 833–834. 20150494 10.1126/science.1186834 PMC3137512

[pbi13419-bib-0013] Flexas, J. and Medrano, H. (2002) Energy dissipation in C3 plants under drought. Funct. Plant Biol. 29, 1209–1215. 32689573 10.1071/FP02015

[pbi13419-bib-0014] Frugoli, J.A. , Zhong, H.H. , Nuccio, M.L. , McCourt, P. , McPeek, M.A. , Thomas, T.L. and McClung, C.R. (1996) Catalase is encoded by a multigene family in *Arabidopsis thaliana* (L.) Heynh. Plant Physiol. 112, 327–336. 8819328 10.1104/pp.112.1.327 PMC157953

[pbi13419-bib-0015] Giri, M.K. , Singh, N. , Banday, Z.Z. , Singh, V. , Ram, H. , Singh, D. *et al* (2017) GBF1 differentially regulates *CAT2* and *PAD4* transcription to promote pathogen defense in *Arabidopsis thaliana* . Plant J. 91, 802–815. 28622438 10.1111/tpj.13608

[pbi13419-bib-0016] Heath, R.L. and Packer, L. (1968) Photoperoxidation in isolated chloroplasts: I. Kinetics and stoichiometry of fatty acid peroxidation. Arch. Biochem. Biophys. 125, 189–198. 5655425 10.1016/0003-9861(68)90654-1

[pbi13419-bib-0017] Horsch, R.B. , Fry, J.E. , Hoffman, N.L. , Eichholtz, D. , Rogers, S.A. and Fraley, R.T. (1985) A simple and general method for transferring genes into plants. Science, 227, 1229–1232. 17757866 10.1126/science.227.4691.1229

[pbi13419-bib-0018] Huang, C. , Xie, Y. and Zhou, X. (2009) Efficient virus‐induced gene silencing in plants using a modified geminivirus DNA1 component. Plant Biotechnol. J. 7, 254–265. 19175519 10.1111/j.1467-7652.2008.00395.x

[pbi13419-bib-0019] Huang, X.S. , Wang, W. , Zhang, Q. and Liu, J.H. (2013) A basic helix‐loop‐helix transcription factor, *PtrbHLH*, of *Poncirus trifoliata* confers cold tolerance and modulates peroxidase‐mediated scavenging of hydrogen peroxide. Plant Physiol. 162, 1178–1194. 23624854 10.1104/pp.112.210740 PMC3668048

[pbi13419-bib-0020] Jaglo‐Ottosen, K.R. , Gilmour, S.J. , Zarka, D.G. , Schabenberger, O. and Thomashow, M.F. (1998) *Arabidopsis CBF1* overexpression induces COR genes and enhances freezing tolerance. Science, 280, 104–106. 9525853 10.1126/science.280.5360.104

[pbi13419-bib-0021] Jefferson, R.A. , Kavanagh, T.A. and Bevan, M.W. (1987) GUS fusions: beta‐glucuronidase as a sensitive and versatile gene fusion marker in higher plants. EMBO J. 6, 3901. 3327686 10.1002/j.1460-2075.1987.tb02730.x PMC553867

[pbi13419-bib-0022] Joo, J. , Lee, Y.H. and Song, S.I. (2014) Rice *CatA*, *CatB*, and *CatC* are involved in environmental stress response, root growth, and photorespiration, respectively. J. Plant Biol. 57, 375–382.

[pbi13419-bib-0023] Juul, T. , Malolepszy, A. , Dybkaer, K. , Kidmose, R. , Rasmussen, J.T. , Andersen, G.R. *et al* (2010) The in vivo toxicity of hydroxyurea depends on its direct target catalase. J. Biol. Chem. 285, 21411–21415. 20452979 10.1074/jbc.M110.103564 PMC2898382

[pbi13419-bib-0024] König, J. , Baier, M. , Horling, F. , Kahmann, U. , Harris, G. , Schürmann, P. and Dietz, K.J. (2002) The plant‐specific function of 2‐Cys peroxiredoxin‐mediated detoxification of peroxides in the redox‐hierarchy of photosynthetic electron flux. Proc. Natl. Acad. Sci. USA, 99, 5738–5743. 11929977 10.1073/pnas.072644999 PMC122841

[pbi13419-bib-0025] Leung, D.W. (2018) Studies of catalase in plants under abiotic stress In Antioxidants and Antioxidant Enzymes in Higher Plants (Gupta D.K., Palma J.M. and Corpas F.J., eds), pp. 27–39. Cham: Springer.

[pbi13419-bib-0026] Li, S.J. , Xie, X.L. , Liu, S.C. , Chen, K.S. and Yin, X.R. (2019) Auto‐and mutual‐regulation between two *CitERFs* contribute to ethylene‐induced citrus fruit degreening. Food Chem. 299, 125163. 31319344 10.1016/j.foodchem.2019.125163

[pbi13419-bib-0027] Licausi, F. , Ohme‐Takagi, M. and Perata, P. (2013) APETALA2/Ethylene Responsive Factor (AP2/ERF) transcription factors: mediators of stress responses and developmental programs. New Phytol. 199, 639–649. 24010138 10.1111/nph.12291

[pbi13419-bib-0028] Lin, R.C. , Park, H.J. and Wang, H.Y. (2008) Role of *Arabidopsis* RAP2.4 in regulating light‐ and ethylene‐mediated developmental processes and drought stress tolerance. Mol. Plant, 1, 42–57. 20031913 10.1093/mp/ssm004

[pbi13419-bib-0029] Liu, Q. , Kasuga, M. , Sakuma, Y. , Abe, H. , Miura, S. , Yamaguchi‐Shinozaki, K. and Shinozaki, K. (1998) Two transcription factors, DREB1 and DREB2, with an EREBP/AP2 DNA binding domain separate two cellular signal transduction pathways in drought‐ and low‐temperature‐responsive gene expression, respectively, in *Arabidopsis* . Plant Cell, 10, 1391–1406. 9707537 10.1105/tpc.10.8.1391 PMC144379

[pbi13419-bib-0030] Liu, W. , Tai, H. , Li, S. , Gao, W. , Zhao, M. , Xie, C. and Li, W.X. (2014) *bHLH122* is important for drought and osmotic stress resistance in *Arabidopsis* and in the repression of ABA catabolism. New Phytol. 201, 1192–1204. 24261563 10.1111/nph.12607

[pbi13419-bib-0031] Liu, Y. , Ji, X. , Nie, X. , Qu, M. , Zheng, L. *et al* (2015) *Arabidopsis* AtbHLH112 regulates the expression of genes involved in abiotic stress tolerance by binding to their E‐box and GCG‐box motifs. New Phytol. 207, 692–709. 25827016 10.1111/nph.13387

[pbi13419-bib-0032] Mhamdi, A. , Queval, G. , Chaouch, S. , Vanderauwera, S. , Van Breusegem, F. and Noctor, G. (2010) Catalase function in plants: a focus on *Arabidopsis* mutants as stress‐mimic models. J. Exp. Bot. 61, 4197–4220. 20876333 10.1093/jxb/erq282

[pbi13419-bib-0033] Miller, G. , Suzuki, N. , Ciftci‐Yilmaz, S. and Mittler, R. (2010) Reactive oxygen species homeostasis and signalling during drought and salinity stresses. Plant, Cell Environ. 33, 453–467. 19712065 10.1111/j.1365-3040.2009.02041.x

[pbi13419-bib-0034] Mittler, R. (2017) ROS are good. Trends Plant Sci. 22, 11–19. 27666517 10.1016/j.tplants.2016.08.002

[pbi13419-bib-0035] Mittler, R. , Vanderauwera, S. , Gollery, M. and Van Breusegem, F. (2004) Reactive oxygen gene network of plants. Trends Plant Sci. 9, 490–498. 15465684 10.1016/j.tplants.2004.08.009

[pbi13419-bib-0036] Mizoi, J. , Shinozaki, K. and Yamaguchi‐Shinozaki, K. (2012) AP2/ERF family transcription factors in plant abiotic stress responses. BBA‐Gene Regul. Mech. 1819, 86–96. 10.1016/j.bbagrm.2011.08.004 21867785

[pbi13419-bib-0037] Moore, K. and Roberts, L.J. (1998) Measurement of lipid peroxidation. Free Radical Res. 28, 659–671. 9736317 10.3109/10715769809065821

[pbi13419-bib-0038] Munné‐Bosch, S. , Schwarz, K. and Alegre, L. (1999) Enhanced formation of α‐tocopherol and highly oxidized abietane diterpenes in water‐stressed rosemary plants. Plant Physiol. 121, 1047–1052. 10557254 10.1104/pp.121.3.1047 PMC59469

[pbi13419-bib-0039] Nakano, T. , Suzuki, K. , Fujimura, T. and Shinshi, H. (2006) Genome‐wide analysis of the ERF gene family in *Arabidopsis* and rice. Plant Physiol. 140, 411–432. 16407444 10.1104/pp.105.073783 PMC1361313

[pbi13419-bib-0040] Niebel, A. , Heungens, K. , Barthels, N. , Inzé, D. , Van, M.M. and Gheysen, G. (1995) Characterization of a pathogen‐induced potato catalase and its systemic expression upon nematode and bacterial infection. Mol. Plant Microbe In. 8, 371–378. 10.1094/mpmi-8-0371 7655060

[pbi13419-bib-0041] Nilson, S.E. and Assmann, S.M. (2007) The control of transpiration. Insights from Arabidopsis. Plant Physiol. 143, 19–27. 17210910 10.1104/pp.106.093161 PMC1761994

[pbi13419-bib-0042] Osakabe, Y. , Osakabe, K. , Shinozaki, K. and Tran, L.S.P. (2014) Response of plants to water stress. Front. Plant Sci. 5, 86. 24659993 10.3389/fpls.2014.00086 PMC3952189

[pbi13419-bib-0043] Polidoros, A.N. , Mylona, P.V. and Scandalios, J.G. (2001) Transgenic tobacco plants expressing the maize *Cat2* gene have altered catalase levels that affect plant‐pathogen interactions and resistance to oxidative stress. Transgenic Res. 10, 555–569. 11817543 10.1023/a:1013027920444

[pbi13419-bib-0044] Rasheed, S. , Bashir, K. , Matsui, A. , Tanaka, M. and Seki, M. (2016) Transcriptomic analysis of soil‐grown *Arabidopsis thaliana* roots and shoots in response to a drought stress. Front. Plant Sci. 7, 180. 26941754 10.3389/fpls.2016.00180 PMC4763085

[pbi13419-bib-0045] Rushton, P.J. , Bokowiec, M.T. , Han, S. , Zhang, H. , Brannock, J.F. , Chen, X. , Laudeman, T.W. *et al* (2008) Tobacco transcription factors: novel insights into transcriptional regulation in the Solanaceae. Plant Physiol. 147, 280–295. 18337489 10.1104/pp.107.114041 PMC2330323

[pbi13419-bib-0046] Sakuma, Y. , Liu, Q. , Dubouzet, J.G. , Abe, H. , Shinozaki, K. and Yamaguchi‐Shinozaki, K. (2002) DNA‐binding specificity of the ERF/AP2 domain of *Arabidopsis* DREBs, transcription factors involved in dehydration‐and cold‐inducible gene expression. Biochem. Biophys. Res. Commun. 290, 998–1009. 11798174 10.1006/bbrc.2001.6299

[pbi13419-bib-0047] Schieber, M. and Chandel, N.S. (2014) ROS function in redox signaling and oxidative stress. Curr. Biol. 24, R453–R462. 24845678 10.1016/j.cub.2014.03.034 PMC4055301

[pbi13419-bib-0048] Shinozaki, K. and Yamaguchi‐Shinozaki, K. (2007) Gene networks involved in drought stress response and tolerance. J. Exp. Bot. 58, 221–227. 17075077 10.1093/jxb/erl164

[pbi13419-bib-0049] Shinozaki, K. , Yamaguchi‐Shinozaki, K. and Seki, M. (2003) Regulatory network of gene expression in the drought and cold stress responses. Curr. Opin. Plant Biol. 6, 410–417. 12972040 10.1016/s1369-5266(03)00092-x

[pbi13419-bib-0050] Smykowski, A. , Zimmermann, P. and Zentgraf, U. (2010) G‐Box binding factor1 reduces CATALASE2 expression and regulates the onset of leaf senescence in *Arabidopsis* . Plant Physiol. 153, 1321–1331. 20484024 10.1104/pp.110.157180 PMC2899923

[pbi13419-bib-0051] Tardieu, F. , Parent, B. , Caldeira, C.F. and Welcker, C. (2014) Genetic and physiological controls of growth under water deficit. Plant Physiol. 164, 1628–1635. 24569846 10.1104/pp.113.233353 PMC3982729

[pbi13419-bib-0052] Thirumalaikumar, V.P. , Devkar, V. , Mehterov, N. , Ali, S. , Ozgur, R. , Turkan, I. *et al* (2017) NAC transcription factor JUNGBRUNNEN1 enhances drought tolerance in tomato. Plant Biotechnol. J. 16, 354–366. 28640975 10.1111/pbi.12776 PMC5787828

[pbi13419-bib-0053] Verslues, P.E. , Agarwal, M. , Katiyar‐Agarwal, S. , Zhu, J. and Zhu, J.K. (2006) Methods and concepts in quantifying resistance to drought, salt and freezing, abiotic stresses that affect plant water status. Plant J. 45, 523–539. 16441347 10.1111/j.1365-313X.2005.02593.x

[pbi13419-bib-0054] Wang, W. , Cheng, Y. , Chen, D. , Liu, D. , Hu, M. , Dong, J. *et al* (2019) The catalase gene family in cotton: genome‐wide characterization and bioinformatics analysis. Cells, 8, 86. 10.3390/cells8020086 PMC6406514 30682777

[pbi13419-bib-0055] Xie, Z. , Nolan, T.M. , Jiang, H. and Yin, Y. (2019) AP2/ERF transcription factor regulatory networks in hormone and abiotic stress responses in *Arabidopsis* . Front. Plant Sci. 10, 228. 30873200 10.3389/fpls.2019.00228 PMC6403161

[pbi13419-bib-0056] Xing, Y. , Jia, W. and Zhang, J. (2007) AtMEK1 mediates stress‐induced gene expression of CAT1 catalase by triggering H_2_O_2_ production in *Arabidopsis* . J. Exp. Bot. 58, 2969–2981. 17728292 10.1093/jxb/erm144

[pbi13419-bib-0057] Yang, H. , Zhao, L. , Zhao, S. , Wang, J. and Shi, H. (2017) Biochemical and transcriptomic analyses of drought stress responses of LY1306 tobacco strain. Scientific Rep. 7, 17442. 10.1038/s41598-017-17045-2 PMC5727203 29234072

[pbi13419-bib-0058] Yao, Y. , He, R.J. , Xie, Q.L. , Zhao, X.H. , Deng, X.M. , He, J.B. *et al* (2017) ETHYLENE RESPONSE FACTOR 74 (ERF74) plays an essential role in controlling a respiratory burst oxidase homolog D (RbohD)‐dependent mechanism in response to different stresses in *Arabidopsis* . New Phytol. 213, 1667–1681. 28164334 10.1111/nph.14278

[pbi13419-bib-0059] Yin, F. , Qin, C. , Gao, J. , Liu, M. , Luo, X. , Zhang, W. *et al* (2015) Genome‐wide identification and analysis of drought‐responsive genes and microRNAs in tobacco. Int. J. Mol. Sci. 16, 5714–5740. 25775154 10.3390/ijms16035714 PMC4394501

[pbi13419-bib-0060] Yoo, C.Y. , Pence, H.E. , Jin, J.B. , Miura, K. , Gosney, M.J. , Hasegawa, P.M. and Mickelbart, M.V. (2010) The *Arabidopsis* GTL1 transcription factor regulates water use efficiency and drought tolerance by modulating stomatal density via transrepression of *SDD1* . Plant Cell, 22, 4128–4141. 21169508 10.1105/tpc.110.078691 PMC3027182

[pbi13419-bib-0061] Zhao, Q. , Ren, Y.R. , Wang, Q.J. , Wang, X.F. , You, C.X. and Hao, Y.J. (2016a) Ubiquitination‐related MdBT scaffold proteins target a bHLH transcription factor for iron homeostasis. Plant Physiol. 172, 1973–1988. 27660166 10.1104/pp.16.01323 PMC5100752

[pbi13419-bib-0062] Zhao, Q. , Ren, Y.R. , Wang, Q.J. , Yao, Y.X. , You, C.X. and Hao, Y.J. (2016b) Overexpression of *MdbHLH104* gene enhances the tolerance to iron deficiency in apple. Plant Biotechnol. J. 14, 1633–1645. 26801352 10.1111/pbi.12526 PMC5066684

[pbi13419-bib-0063] Zhou, Y.B. , Liu, C. , Tang, D.Y. , Yan, L. , Wang, D. , Yang, Y.Z. *et al* (2018) The receptor‐like cytoplasmic kinase STRK1 phosphorylates and activates CatC, thereby regulating H_2_O_2_ homeostasis and improving salt tolerance in rice. Plant Cell, 30, 1100–1118. 29581216 10.1105/tpc.17.01000 PMC6002193

[pbi13419-bib-0064] Zhu, J.K. (2016) Abiotic stress signaling and responses in plants. Cell, 167, 313–324. 27716505 10.1016/j.cell.2016.08.029 PMC5104190

[pbi13419-bib-0065] Zimmermann, P. , Heinlein, C. , Orendi, G. and Zentgraf, U. (2006) Senescence‐specific regulation of catalases in *Arabidopsis thaliana* (L.) Heynh. Plant, Cell Environ. 29, 1049–1060. 17080932 10.1111/j.1365-3040.2005.01459.x

[pbi13419-bib-0066] Zou, J.J. , Li, X.D. , Ratnasekera, D. , Wang, C. , Liu, W.X. , Song, L.F. *et al* (2015) *Arabidopsis* CALCIUM‐DEPENDENT PROTEIN KINASE8 and CATALASE3 function in abscisic acid‐mediated signaling and H_2_O_2_ homeostasis in stomatal guard cells under drought stress. Plant Cell, 27, 1445–1460. 25966761 10.1105/tpc.15.00144 PMC4456645

