# Supplementary material for: The AP2 transcription factor NtERF172 confers drought resistance by modifying NtCAT
Source: Plant Biotechnol J. 2020 Jun 15;18(12):2444–55. doi: 10.1111/pbi.13419 (PMC7680539; doi:10.1111/pbi.13419)
Supplement: Supplementary file 1 — Figure S1. Promoter analysis of the NtCAT promoter. Figure S2. Phylogenetic and conserved domain analysis of NtERF172. Figure S3. Subcellular localization and transcriptional activity of NtERF172. Figure S4. Analysis of germination of wild‐type (WT) and NtERF172‐ox plants exposed to PEG6000 treatment. Figure S5. Analysis of relative water content (RWC), ion leakage (IL), malondialdehyde (MDA), and fresh weight (FW) in wild‐type (WT) and NtERF172‐ox plants under normal and drought conditions. Figure S6. Expression analysis of the NtERF172 and NtCAT genes. Figure S7. Oxidative stress tolerance assays in 2mDNA1 and 2mDNA1::NtERF172 transgenic plants treated with 5% H2O2. Table S1. Primers used for ChIP‐PCR, Y1H assays, transient expression assays and plant transformation. Table S2. Primers used in real‐time qRT‐PCR. [file PBI-18-2444-s001.docx]

The AP2 transcription factor *NtERF172* confers drought resistance by modifying *NtCAT* – Supplementary material

Qiang Zhao^1,^*, Ri-Sheng Hu^2^, Dan Liu^3^, Xin Liu^1^, Jie Wang^3^, Xiao-Hua Xiang^4,^*, Yang-Yang Li^2,^*

^1^College of Horticulture, Qingdao Agricultural University, Qingdao 266109, China

^2^Hunan Tobacco Research Institute, Changsha 410004, Hunan, China;

^3^Tobacco Research Institute, Chinese Academy of Agricultural Sciences, Qingdao 266101, Shandong Province, China;

^4^Haikou Cigar Research Institution, Haikou, Hainan Province, China;

***Author for correspondence:**

(1) Qiang Zhao

Address: College of Horticulture, Qingdao Agricultural University, Qingdao 266109, China

E-mail: zhaoqiang000666@163.com

(2) Xiao-Hua Xiang

Address: Hainan Cigar Research Institution, Haikou, Hainan Province, China

E-mail: xiangxiaohuacaas@163.com

(3) Yang-Yang Li

Address: Hunan Tobacco Research Institute, Changsha 410004, Hunan, China

E-mail: shen-ly@163.com


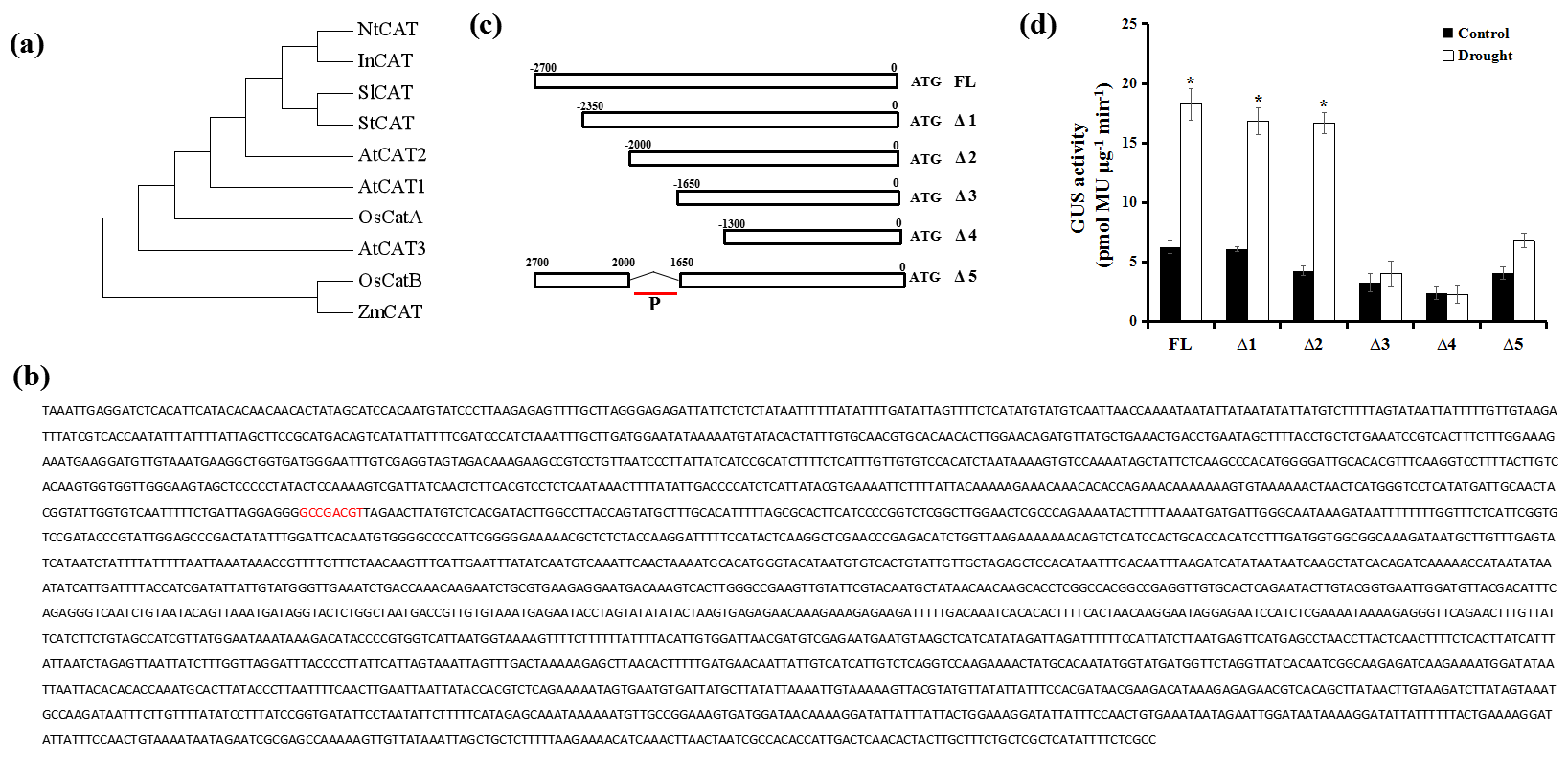


**Figure S1.** Promoter analysis of the *NtCAT* promoter.

(a) Phylogenetic analysis of the catalases (CAT) proteins from different species. The species of origin of the CATs are indicated by the abbreviation before the gene names: At, *Arabidopsis thaliana*; In, *Ipomoea nil*; Os, *Oryza sativa*; Sl, *Solanum lycopersicum*; St, *Solanum tuberosum*; Zm, *Zea mays*. (b) The promoter sequence of *NtCAT* is shown. The drought-responsive element (DRE) motif is red. (c) A diagram showing six deletion constructs of the *NtCAT* promoter. The length of the promoter for each construct is indicated on the left, and the name of the construct is indicated on the right. (d) β-glucuronidase (GUS) activity of a representative line for each P*_NtCAT_::GUS* construct in the control or drought conditions. Values are means ± SD of three biological replicates. Asterisk indicates a significant difference relative to WT (*P < 0.01).


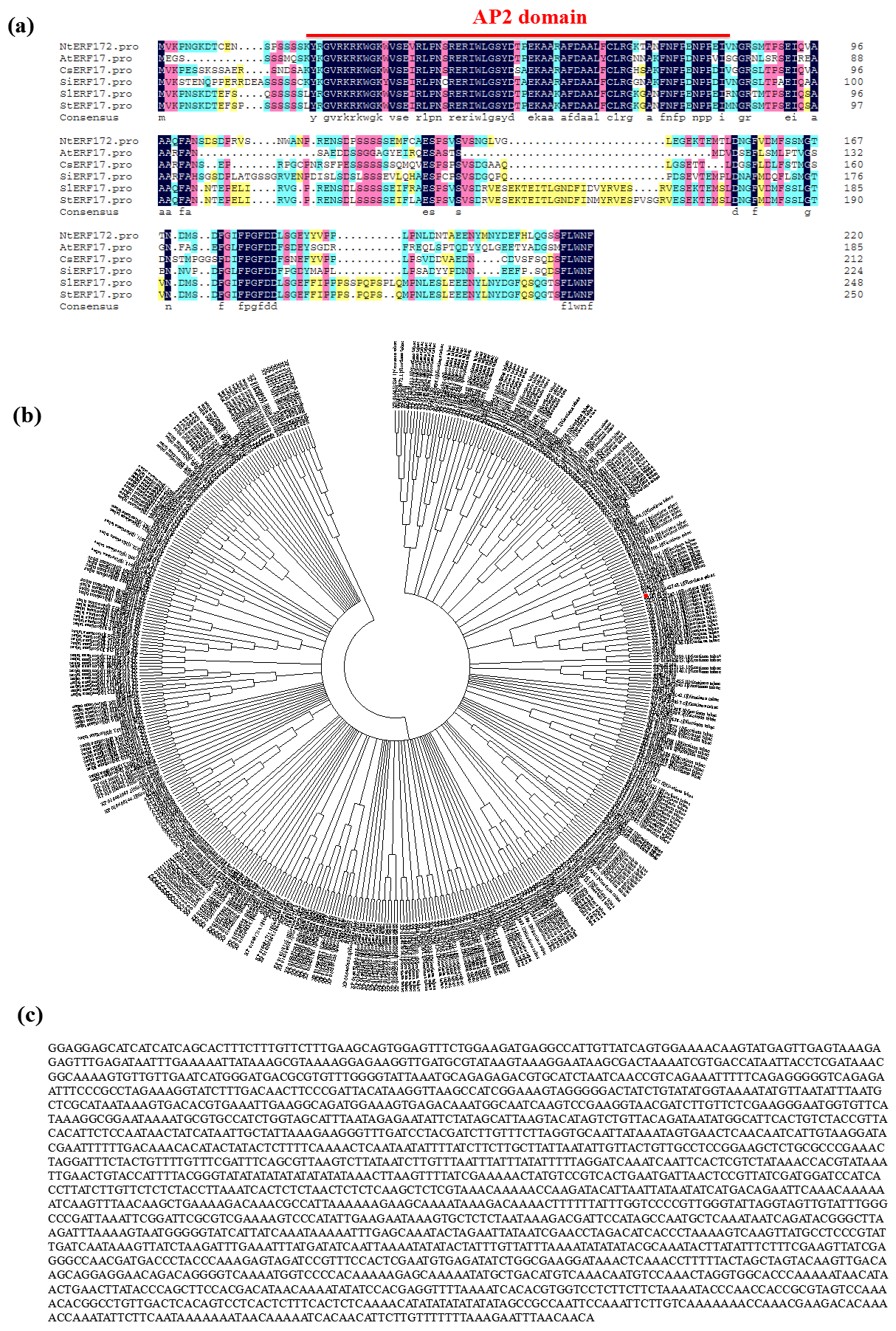


**Figure S2.** Phylogenetic and conserved domain analysis of NtERF172.

(a) The AP2 domain sequences are highly conserved across NtERF172 from tobacco and other plant species (CsERF17, XP_028121981.1; SiERF17, XP_011093871.1; SlERF17, XP_004251702.1; StERF17, XP_006345254.1;). (b) The phylogenetic tree constructed using tobacco AP2/ERFs and other plant species. NtERF172 is shown by a red circle. (c) The promoter sequence of *NtERF172* is shown.


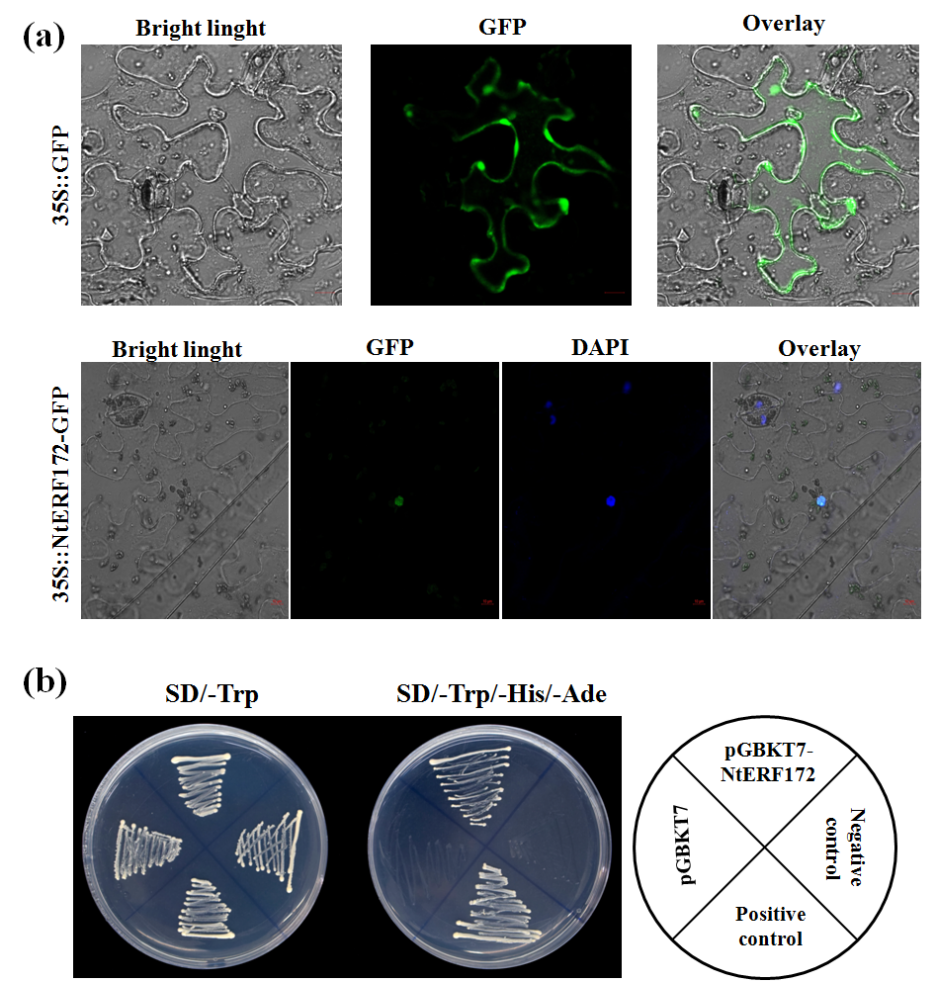


**Figure S3.** Subcellular localization and transcriptional activity of NtERF172.

(a) Subcellular localization of NtERF172. The *35S::GFP* and *35S::NtERF172-GFP* fusion construct was transformed into *N. benthamiana* epidermal cells. Images were taken under bright light and green fluorescent protein (GFP) fluorescence. 4ʹ,6-Diamidino-2-phenylindole (DAPI) was used to stain the nuclei. The overlaid images are shown on the right. Bar = 10 µm.

(b) The yeast cells transformed with different constructs on the SD/-Trp and SD/-Trp/-His/-Ade medium.


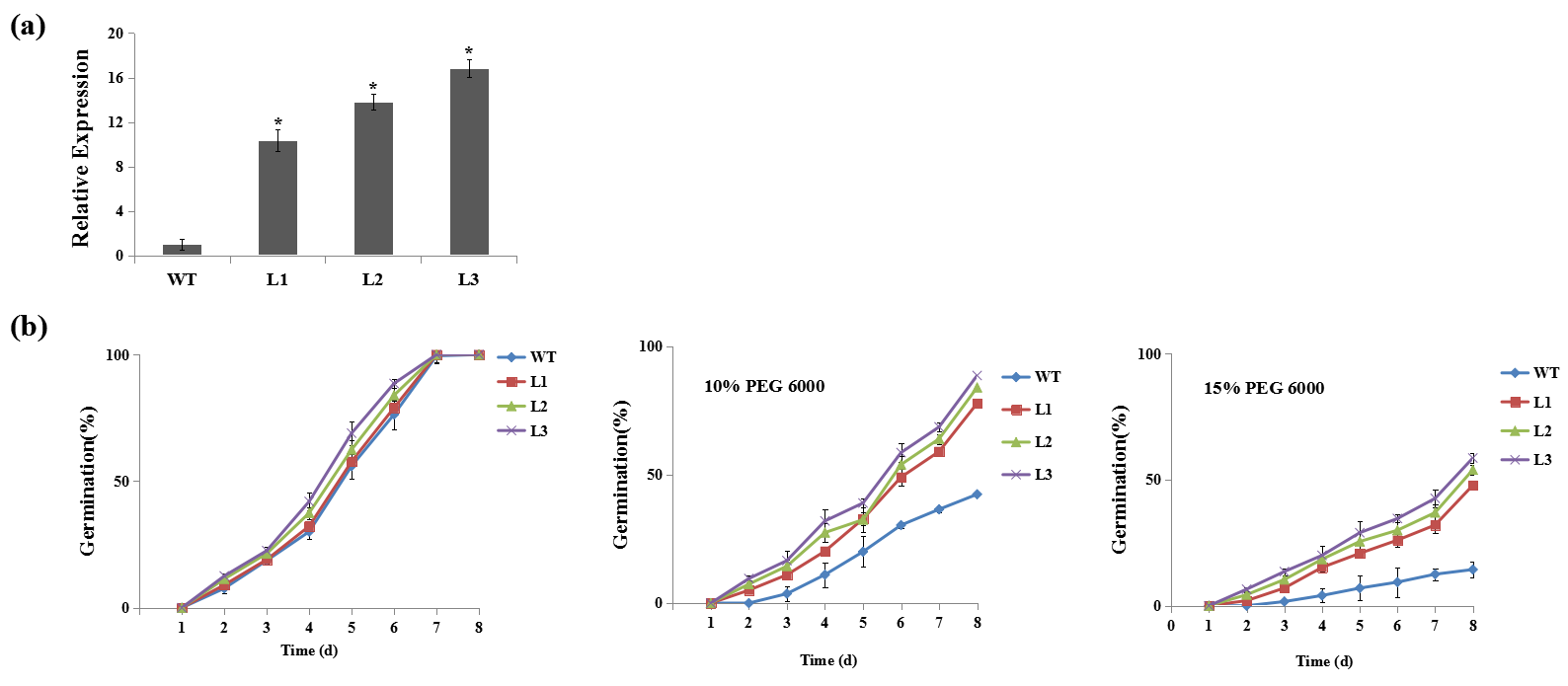


**Figure S4.** Analysis of germination of wild-type (WT) and *NtERF172-*ox plants exposed to PEG6000 treatment.

(a) Expression of *NtERF172* in the transgenic lines. The *NtActin* gene was used as the internal control for normalization. Values are means ± SD of three biological replicates. Asterisk indicates a significant difference relative to WT (*P < 0.01). (c) Germination analysis of wild-type (WT) and *NtERF172-*ox plant seeds after treatment with 10% or 15% PEG 6000. The data represent the means ± SD from at least three biological replicates.

**
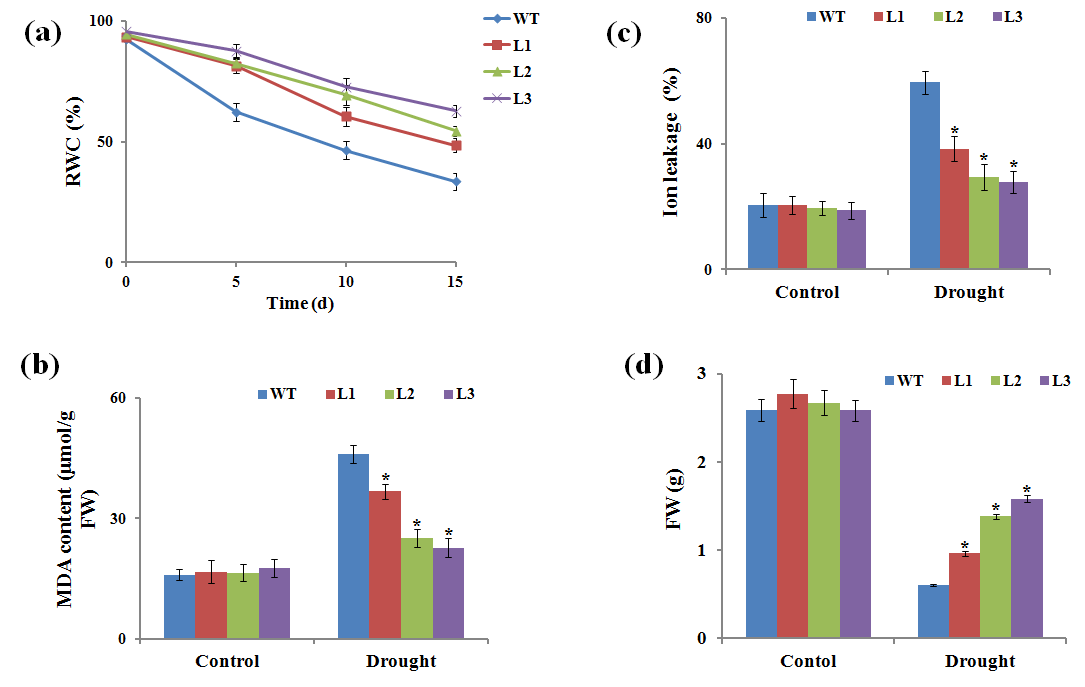
**

**Figure S5.** Analysis of (a) relative water content (RWC), (b) ion leakage (IL), (c) malondialdehyde (MDA), and (d) fresh weight (FW) in wild-type (WT) and *NtERF172-*ox plants under normal and drought conditions.

Thirty-day-old tobacco plants were deprived of water. Then tobacco leaves were collected to measure RWC, IL, MDA content, and FW. Data represent means ± SD calculated from at least three biological replicates. Asterisk indicates a significant difference relative to WT (*P < 0.01).


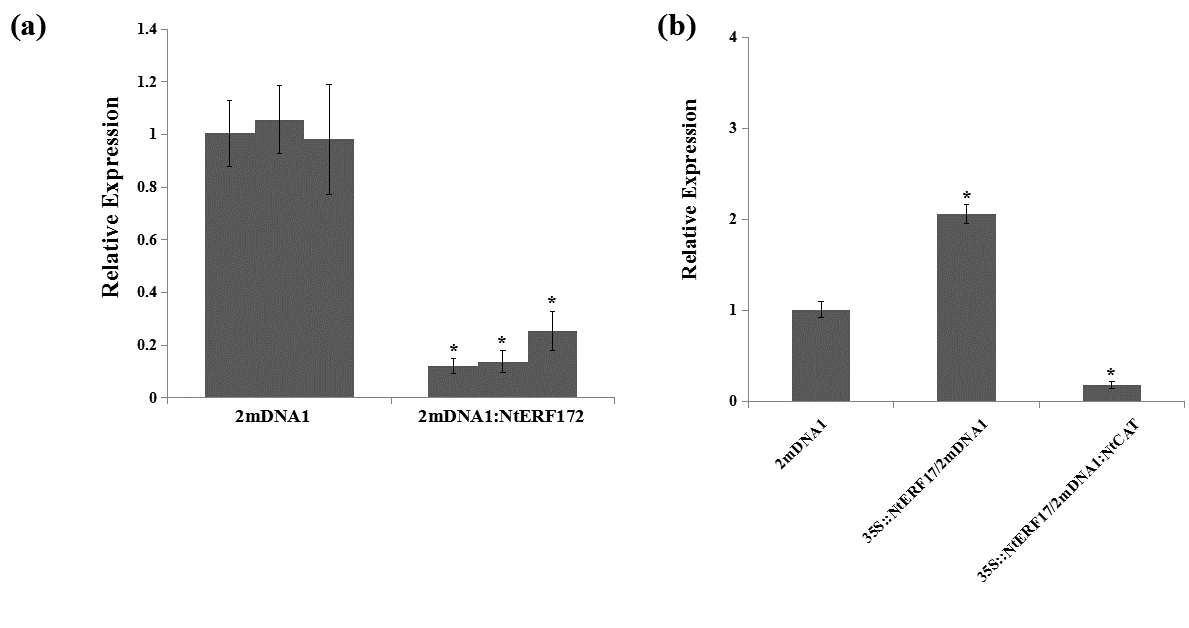


**Figure S6.** Expression analysis of the *NtERF172* and *NtCAT* genes.

1. Analysis of *NtERF172* transcript level in 2mDNA1 and *NtERF172*-silenced (2mDNA1:NtERF172) plants. Three biological replicates were tested and yielded similar results. (b) Expression levels of *NtCAT* in 2mDNA1, *NtERF172*-ox/2mDNA1 and *NtERF172*-ox/2mDNA1:NtCAT plants by qRT-PCR. Data represent means ± SD calculated from three biological replicates. Asterisk indicates a significant difference relative to 2mDNA1 (*P < 0.01).


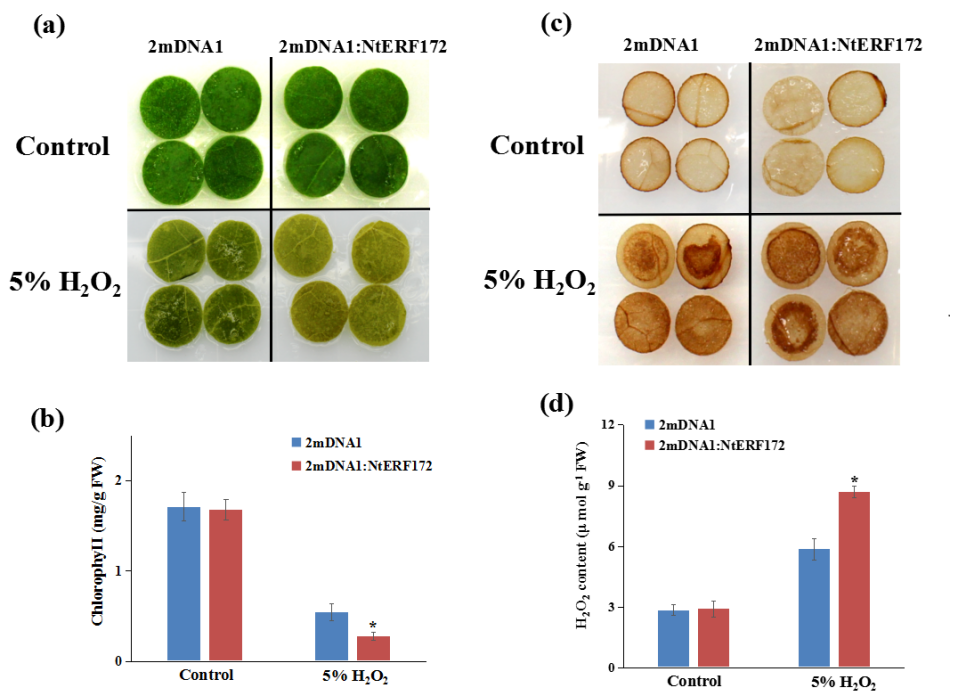


**Figure S7.** Oxidative stress tolerance assays in 2mDNA1 and 2mDNA1::NtERF172 transgenic plants treated with 5% H_2_O_2_.

(a) The phenotype in leaf pieces of 2mDNA1 and 2mDNA1*::*NtERF172 plants after 5% H_2_O_2_ treatment. (b) Chl content in leaf pieces of 2mDNA1 and 2mDNA1*::*NtERF172 transgenic plants. FW, Fresh weight. (c, d) DAB staining and H_2_O_2_ contents of leaf pieces from 2mDNA1 and 2mDNA1*::*NtERF172 plants under control or 5% H_2_O_2_ treatment conditions. Data represent means ± SD calculated from three biological replicates. Three biological experiments produced similar results. Asterisk indicates a significant difference relative to 2mDNA1 (*P < 0.01).

**Table S1** Primers used for ChIP-PCR, Y1H assays, transient expression assays and plant transformation.

| Gene |  | Primers |
| --- | --- | --- |
| *NtCAT-P* | | 5’-TAAATTGAGGATCTCACATTC-3’ |
|  |  | 5’-GGCGAGAAAATATGAGCGAGC-3’ |
| *NtCAT-P1* | | 5’-GGGGCCGACGTTAGAACTTA-3’ |
|  |  | 5’-ATAGTCGGGCTCCAATACGG-3’ |
| *NtCAT-mP1* | | 5’-GGGACCCGGGTTAGAACTTA-3’ |
|  |  | 5’- ATAGTCGGGCTCCAATACGG -3’ |
| *NtCAT-*ox | | 5’-ATGATGCAGAATCGCCCTTC-3’ |
|  |  | 5’-CATTGTGGGCCTTACATT-3’ |
| 2mDNA1:NtCAT | | 5’-CCTGAGGACATCTTGCCACT-3’ |
|  |  | 5’-TAACGGGGAGCTGCAGATAG-3’ |
| *NtERF172-*ox | | 5’-ATGGTGAAACCCAACGGA-3’ |
|  |  | 5’-AAAGTTCCAAAGAAAAGAAGATCC-3’ |
| 2mDNA1:NtERF172 | | 5’-AGACTACCAAACAGCAGGGA-3’ |
|  |  | 5’-GCCCAATTACTAACCCGAGG-3’ |
| *NtCAT-P-*∆1 | | 5’-CCTGAATAGCTTTTACCTGC-3’ |
|  |  | 5’-GGCGAGAAAATATGAGCGAGC-3’ |
| *NtCAT-P-*∆2 | | 5’-AAAATTCTTTTATTACAAAAAGAAAC-3’ |
|  |  | 5’-GGCGAGAAAATATGAGCGAGC-3’ |
| *NtCAT-P-*∆3 | | 5’-AAAACGCTCTCTACCAAGGA-3’ |
|  |  | 5’-GGCGAGAAAATATGAGCGAGC-3’ |
| *NtCAT-P-*∆4 | | 5’-ATATTATTGTATGGGTTGAA-3’ |
|  |  | 5’-GGCGAGAAAATATGAGCGAGC-3’ |

**Table S2** Primers used in real time qRT-PCR.

| Gene |  | Primers |
| --- | --- | --- |
| *NtActin* | | 5’-CTATTCTCCGCTTTGGACTTGGCA-3’ |
|  |  | 5’-ACCTGCTGGAAGGTGCTGAGGGAA-3’ |
| *NtCAT* | | 5’-GCCAAACTATCTGCAGCTCC-3’ |
|  |  | 5’-TCACGCTTCCCTGTCAAGAT-3’ |
| *NtERF172* | | 5’-CGCCGTCGGAAATTCAAGTA-3’ |
|  |  | 5’-TCCAGGGTCATTTCCGTCTT-3’ |
